# The M-phase regulatory phosphatase PP2A-B55δ opposes protein kinase A on Arpp19 to initiate meiotic division

**DOI:** 10.1038/s41467-021-22124-0

**Published:** 2021-03-23

**Authors:** Tom Lemonnier, Enrico Maria Daldello, Robert Poulhe, Tran Le, Marika Miot, Laurent Lignières, Catherine Jessus, Aude Dupré

**Affiliations:** 1 Sorbonne Université, CNRS, Laboratoire de Biologie du Développement—Institut de Biologie Paris Seine, LBD—IBPS, Paris, France; 2 Université de Paris, CNRS, Institut Jacques Monod, Paris, France

**Keywords:** Kinases, Meiosis, Phosphorylation, Xenopus, Oogenesis

## Abstract

Oocytes are held in meiotic prophase for prolonged periods until hormonal signals trigger meiotic divisions. Key players of M-phase entry are the opposing Cdk1 kinase and PP2A-B55δ phosphatase. In *Xenopus*, the protein Arpp19, phosphorylated at serine 67 by Greatwall, plays an essential role in inhibiting PP2A-B55δ, promoting Cdk1 activation. Furthermore, Arpp19 has an earlier role in maintaining the prophase arrest through a second serine (S109) phosphorylated by PKA. Prophase release, induced by progesterone, relies on Arpp19 dephosphorylation at S109, owing to an unknown phosphatase. Here, we identified this phosphatase as PP2A-B55δ. In prophase, PKA and PP2A-B55δ are simultaneously active, suggesting the presence of other important targets for both enzymes. The drop in PKA activity induced by progesterone enables PP2A-B55δ to dephosphorylate S109, unlocking the prophase block. Hence, PP2A-B55δ acts critically on Arpp19 on two distinct sites, opposing PKA and Greatwall to orchestrate the prophase release and M-phase entry.

## Abstract (web summary)

Mechanisms triggering meiotic divisions of oocytes remain unclear. Here, the authors report that meiosis resumption relies on the timely phosphorylation of Arpp19 protein at two distinct sites, which depends on two kinases (PKA and Gwl) and a single phosphatase (PP2A-B55δ).

## Introduction

In vertebrates, oocyte meiotic divisions are controlled by the cAMP-dependent protein kinase A, PKA. The activity of PKA is responsible for arresting fully-grown oocytes in prophase of the first meiotic division for long-lasting periods in order to allow cell growth and nutrient accumulation required for fertilization and the early embryogenesis^1,2^. The post-transcriptional mechanisms controlled by PKA in oocytes were largely unknown until the identification of the protein Arpp19 as its long sought substrate in *Xenopus*^3^. In this species, Arpp19 phosphorylation at S109 by PKA is critical to maintain oocytes arrested in prophase. Release from the prophase block is promoted by progesterone, which triggers within 0.5 h a drop in the cAMP concentration and the down-regulation of PKA activity. As a result, Arpp19 is dephosphorylated at S109 and unlocks a signaling pathway that leads within 3 to 5 h to the activation of the Cdk1-Cyclin B complex and Greatwall kinase (Gwl), or MPF (M-phase promoting factor), the universal inducer of eukaryotic cell division^4^. Once activated, Cdk1-Cyclin B complexes trigger the resumption of the first meiotic cell division which starts with nuclear envelope breakdown, termed germinal vesicle breakdown (GVBD) in oocytes^5^.

The biochemical steps linking PKA downregulation and Arpp19 dephosphorylation at S109 to Cdk1-Cyclin B activation involve the translation of two proteins, Cyclin B and the kinase Mos^6^. Newly synthesized Cyclin B associates with free Cdk1 while Mos indirectly activates MAPK (mitogen-activated protein kinase). Both events allow the formation of newly active Cdk1-Cyclin B complexes and the hyperphosphorylation of the two opposed regulators of Cdk1, the phosphatase Cdc25 and the kinase Myt1^5^. Upon phosphorylation, Cdc25 activates Cdk1 by dephosphorylating T14 and Y15, whereas its antagonistic enzyme, Myt1, is concomitantly inhibited^7^. Importantly, Cdk1 activation also requires the inhibition of a specific phosphatase, the PP2A-B55δ isoform, which counteracts Cdk1-dependent phosphorylations of mitotic/meiotic substrates, likely including Cdc25 and Myt1^8–12^. PP2A-B55δ inhibition is achieved by Arpp19, a specific inhibitor of this phosphatase when phosphorylated at S67 by Gwl^13,14^. Hence, Arpp19 is a central player of meiosis resumption through two distinct functions at two different periods. In prophase oocytes, Arpp19 is phosphorylated by PKA at S109 and holds the prophase block. Upon hormonal stimulation, its dephosphorylation at S109 occurs within 1 h and unlocks a signaling pathway leading to meiotic division. At the very end of this molecular pathway, 3–5 h later, Arpp19 is phosphorylated at S67 by Gwl and inactivates PP2A-B55δ, hence promoting Cdk1-Cyclin B activation^5^.

The molecular regulation of this last step, involving Gwl, Arpp19 and PP2A, has been well deciphered in mitosis and meiosis. PP2A is a major S/T protein phosphatase conserved across eukaryotes. It acts as a heterotrimeric complex composed of a catalytic C subunit, a scaffolding A subunit, and a variable B subunit, which regulates PP2A intracellular localization and substrate specificity^15^. Eukaryotes have four B-subunit families known as B (B55), B’ (B56), B” (PR72) and B”' (striatin), each of them comprising several very close isoforms whose number differs according the species^16^. From yeast to humans, Gwl kinase displays conserved functions in mitosis^17^, which are mediated by the phosphorylation of its sole substrate identified so far, Arpp19, and its paralog, α-endosulfine (ENSA). ENSA and Arpp19 derive from the duplication of an ancestral gene that evolved through new duplications and losses (http://www.ensembl.org; http://www.treefam.org), providing from one to four homologs in the different species^18,19^. Both Arpp19 and ENSA share highly conserved sequence identity/similarity across most eukaryotes. Their most conserved region is centered around the FD**S**_**67**_GDY motif (*X. laevis* numbering). Within this motif, S67 is phosphorylated by Gwl to the same extent in Arpp19 and ENSA, generating phosphorylated proteins that bind to and equally inhibit the specific PP2A-B55δ isoform by titrating the phosphatase away from all other substrates and making themselves its preferential substrates^20,21^. Whether Arpp19 and ENSA display specific functions is not clear, although some evidence shows that, unlike ENSA, Arpp19 plays an essential role during mouse embryogenesis and in regulating mitotic and meiotic divisions^22^. In *Xenopus* oocyte, it is clearly established that S67 phosphorylation of Arpp19 by Gwl promotes its binding to PP2A-B55δ and the inhibition of the phosphatase^23,24^. Released from the activity of its opposite enzyme, Cdk1 phosphorylates its two antagonistic regulators, Cdc25 and Myt1, setting up the positive feedback loop responsible for its abrupt and full activation^5^. Importantly, the activation of the Gwl/Arpp19/PP2A-B55δ module depends on Cdk1 activity^24–27^, positioning this module inside the auto-activation loop. Hence, the antagonistic relationship between Arpp19-Gwl and PP2A-B55δ greatly contributes to the abruptness and irreversibility of cell division entry^28^.

PKA phosphorylates ENSA and Arpp19 at a consensus RKP/S**S**_**109**_LV motif (*X. laevis* numbering) conserved among most animals. Specific functions have been attributed to the PKA-phosphorylated form of Arpp19/ENSA, notably in striatal neurons upon dopaminergic stimulation^29^. No specific role related to cell division had been described until we discovered that Arpp19 phosphorylation by PKA is essential to arrest *Xenopus* oocytes in prophase^3^. The S109 phosphorylation by PKA does not impede the phosphorylation at S67 by Gwl nor its ability to inhibit PP2A-B55δ when phosphorylated at S67^26^. Moreover, Arpp19 is rephosphorylated at S109 by an unknown kinase distinct from PKA, concomitantly with its S67 phosphorylation by Gwl, at time of Cdk1 activation^3^. Thus, the events controlled by the S109 phosphorylation of Arpp19 that maintain the prophase block in oocytes remain an open question. Another key issue to unravel the prophase release regards the identity of the phosphatase that dephosphorylates Arpp19 at S109 at the onset of meiosis resumption. Since this event is important to unlock the transduction pathway leading to cell division, this unidentified phosphatase is a critical player of oocyte meiotic division.

Here, we identify PP2A-B55δ as the phosphatase that dephosphorylates Arpp19 at S109, thus enabling oocytes to resume meiosis. The level of Arpp19 phosphorylated at S109 in prophase-arrested oocytes results from a balance between PKA and PP2A-B55δ activities in favor of the kinase. Upon hormonal stimulation, PP2A-B55δ activity remains unchanged while PKA is downregulated, leading to the partial dephosphorylation of Arpp19 at S109 that unlocks the prophase arrest. Therefore, the timing of meiosis resumption relies on the temporal coordination of S109 and S67 phosphorylations of Arpp19, orchestrated by one single phosphatase, PP2A-B55δ, opposing two kinases, PKA and Gwl.

## Results

### Active Arpp19 dephosphorylation at S109 opposed by PKA in prophase oocytes

**Fig. 1:**
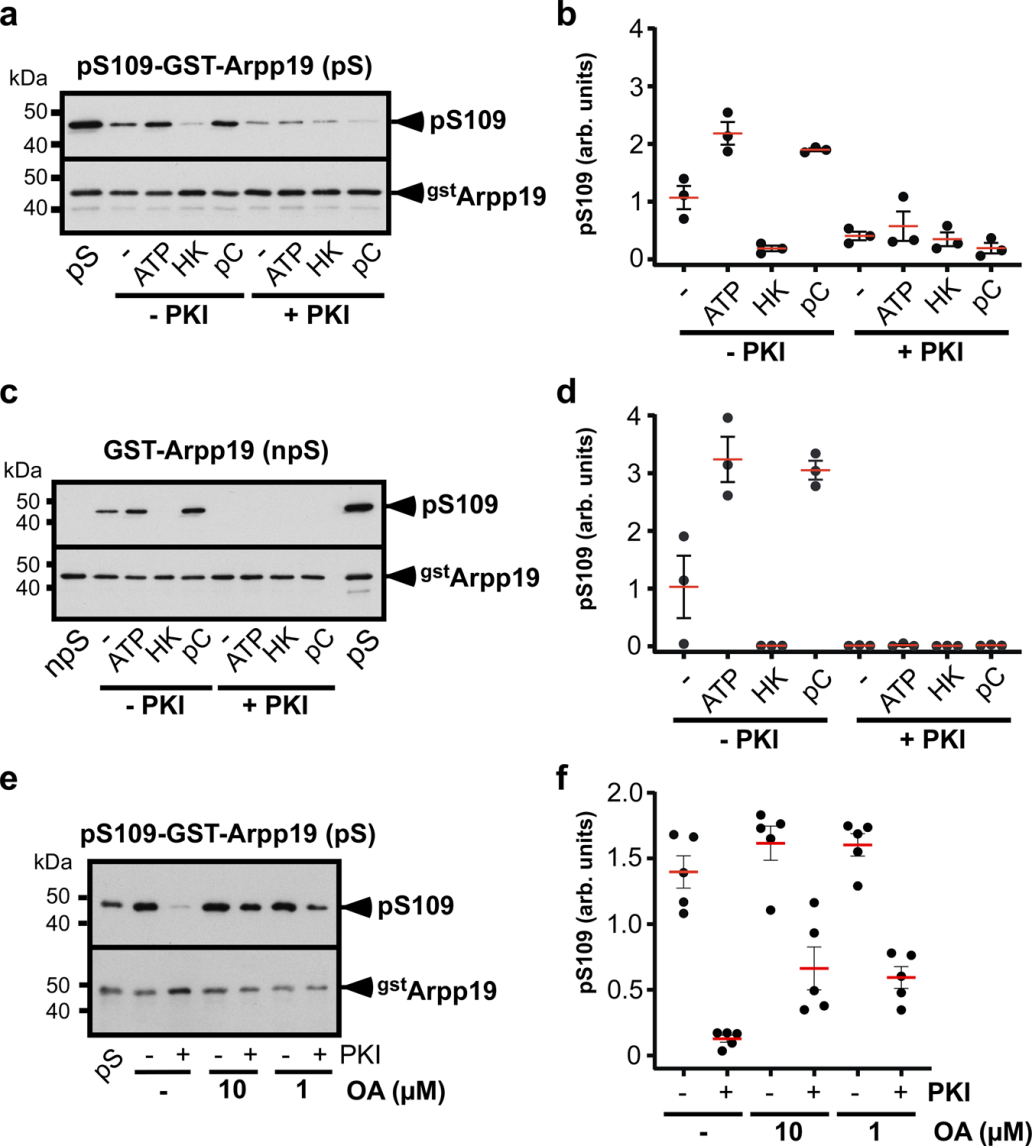
S109-phosphatase is active, OA-sensitive and counterbalanced by PKA in prophase extracts. **a**–**d** Extracts from prophase oocytes were supplemented or not with either ATP or hexokinase and glucose (HK), in the presence or not of phosphocreatine (pC). Extracts were then incubated or not with PKI and either pS109-GST-Arpp19 (pS: phosphorylated substrate) (**a**–**b**) or GST-Arpp19 (npS: non-phosphorylated substrate) (**c**–**d**). S109 phosphorylation of GST-Arpp19 (pS109) and total GST-Arpp19 (^gst^Arpp19) were analyzed by western blot with phospho-S109-Arpp19 and GST antibodies. S109-phosphatase activity: one representative experiment (**a**) and quantifications of S109 phosphorylation from 3 independent experiments (**b**). S109-kinase activity: one representative experiment (**c**) and quantifications of S109 phosphorylation from 3 independent experiments (**d**). **e**–**f** Prophase extracts were incubated or not with 1 μM or 10 μM okadaic acid (OA), supplemented or not with PKI and further incubated with pS109-GST-Arpp19 (pS) in the presence of ATP. S109 phosphorylation of GST-Arpp19 (pS109) and total GST-Arpp19 (^gst^Arpp19) were analyzed as in panel (**a**). One representative experiment and quantifications of S109 phosphorylation from 5 independent experiments are presented in (**e**) and (**f**) respectively. For quantifications, data are shown as mean (red bars) ± SEM. Each dot represents one experiment. arb. units: arbitrary units. kDa: kiloDalton. Source data are provided as a Source Data file.

The S109 residue of Arpp19 phosphorylated by PKA in prophase oocytes is dephosphorylated in response to progesterone by an unknown phosphatase^3^, termed S109-phosphatase until its identification. The level of S109-phosphorylated Arpp19 in prophase-arrested oocytes could result from either the sole activity of PKA or a balance between PKA and S109-phosphatase in favor of PKA. To address this issue, we first assayed S109-phosphatase activity in extracts from prophase oocytes. As a substrate, we used GST-tagged Arpp19 previously in vitro phosphorylated at S109 by PKA (pS109-GST-Arpp19)^26^. Note that GST-Arpp19 is partially proteolyzed during either its expression in bacteria or its purification, occasionally producing a band of lower molecular weight than the full-length protein that lacks S109 but is recognized by the anti-GST antibody (Supplementary Fig. 1). pS109-GST-Arpp19 was coupled to GSH-beads and then incubated in prophase extracts. S109 phosphorylation of pS109-GST-Arpp19 recovered from extracts was monitored by western blot using a specific phospho-S109-Arpp19 antibody^3^. Arpp19 was efficiently dephosphorylated at S109 (Fig. 1a and b), showing that S109-phosphatase is active in prophase extracts. Oocyte lysis leads to ATP hydrolysis and as a result, oocyte extracts contain low levels of ATP that prevent kinases from functioning. Interestingly, adding ATP reduced Arpp19 dephosphorylation at S109 (Fig. 1a and b). To control the ATP amount, prophase extracts were supplemented with hexokinase, which fully depletes ATP^30^. Under this condition, Arpp19 was strongly dephosphorylated at S109 (Fig. 1a and b). In contrast, in the presence of phosphocreatine that replenishes ATP^30^, Arpp19 dephosphorylation at S109 was severely impaired (Fig. 1a and b). Altogether, these results suggest that a kinase counteracts S109-phosphatase. When the specific inhibitor of PKA, PKI^31^, was added to extracts in the presence of ATP, pS109-GST-Arpp19 was efficiently dephosphorylated (Fig. 1a and b). This indicates that S109-phosphatase activity is counterbalanced by PKA in prophase extracts. Furthermore, non-phosphorylated GST-Arpp19 was efficiently phosphorylated at S109 in extracts, a process that was dependent on ATP and sensitive to PKI (Fig. 1c and d). These results demonstrate that PKA and S109-phosphatase are both active and regulate Arpp19 phosphorylation at S109 in prophase extracts, with PKA dominating. In subsequent experiments, PKI was included in order to block PKA activity and to facilitate analysis of its opposing phosphatase.

We next determined whether S109-phosphatase belongs to the group of okadaic acid (OA)-sensitive phosphatases, which includes PP1, PP2A and phosphatases related to PP2A^16,32^. Prophase extracts were first supplemented with 1 or 10 µM OA. PKI and the S109 phosphatase substrate, pS109-GST-Arpp19, were then added in the presence of ATP. Both OA concentrations prevented Arpp19 dephosphorylation at S109 (Fig. 1e and f). Hence, S109-phosphatase is an OA-sensitive phosphatase that is active and antagonizes PKA in prophase extracts.

### S109-phosphatase is distinct from PP1 and PP5

**Fig. 2:**
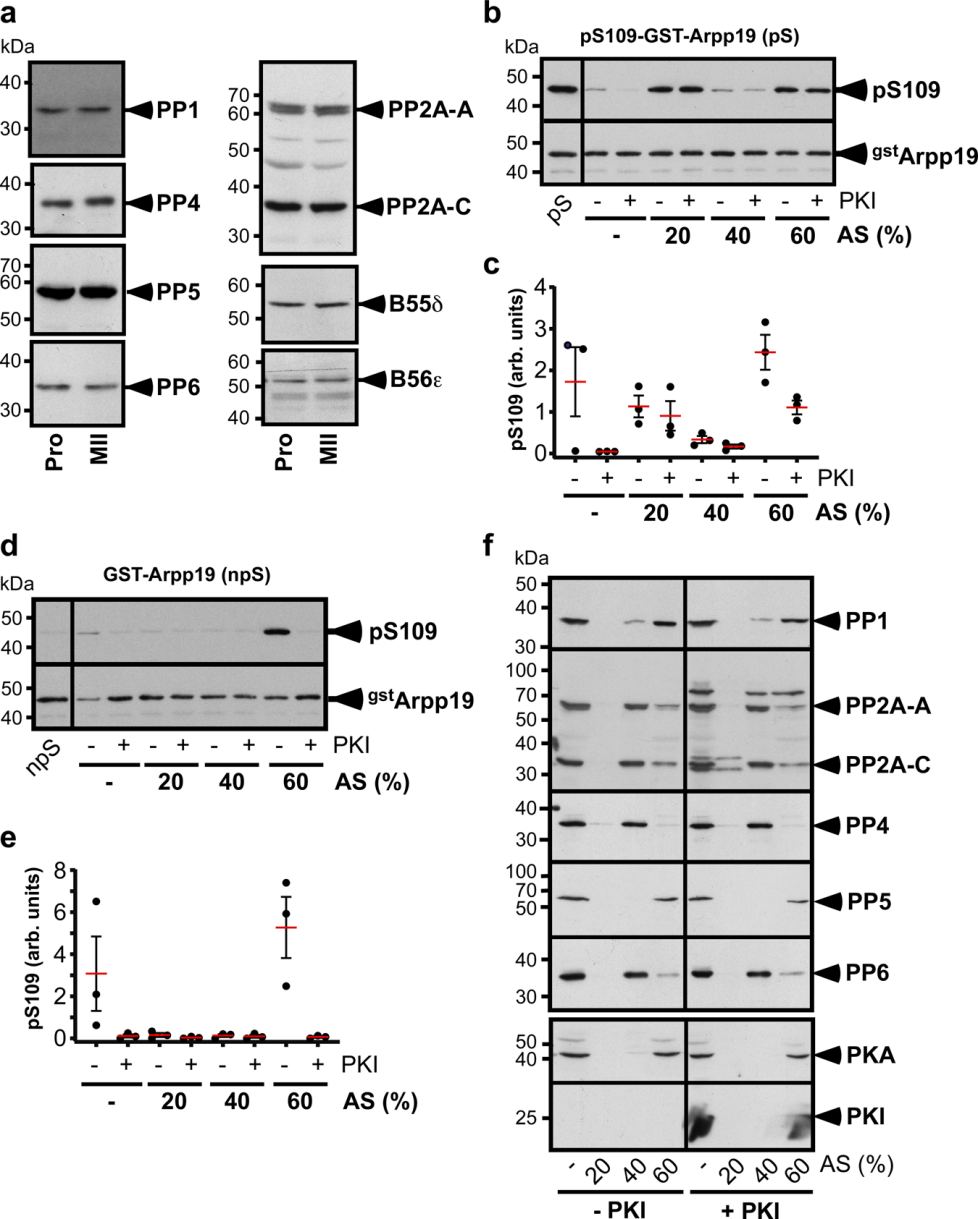
Ammonium sulfate precipitation separates S109-phosphatase from PKA, PP1 and PP5. **a** Western blot analysis of various S/T phosphatases sensitive to OA in lysates from prophase (Pro) or metaphase II (MII) oocytes using specific antibodies directed against catalytic subunits of PP1, PP2A (PP2A-C), PP4, PP5, PP6, and PP2A-regulatory subunit A (PP2A-A), B55δδ, and B56ε. The experiment was repeated 3 times with similar results. **b**–**e** Prophase extracts supplemented or not with PKI were precipitated by serial addition of ammonium sulfate (AS) as indicated. (–): Starting extracts without AS. Pellets were recovered and used for enzymatic assays and western blots with phospho-S109-Arpp19 and GST antibodies. S109-phosphatase activity was assayed using pS109-GST-Arpp19 (pS: phosphorylated substrate): one representative experiment (**b**) and quantifications of S109 phosphorylation from 3 independent experiments (**c**). **d**–**e** PKA activity was assayed using GST-Arpp19 (npS: non-phosphorylated substrate): one representative experiment (**d**) and quantifications of S109 phosphorylation from 3 independent experiments (**e**). For quantifications, data are presented as mean (red bars) ± SEM. Each dot represents one experiment. arb. units: arbitrary units. **f** Western blot analysis of initial extracts (–) and AS precipitates using specific antibodies directed against catalytic subunits of PP1, PP2A (PP2A-C), PP4, PP5, PP6, PKA, and against PP2A scaffold subunit A (PP2A-A) and PKI. The experiment was repeated 3 times with similar results. kDa: kiloDalton. Source data are provided as a Source Data file.

As the S109-phosphatase is inhibited by OA, we analyzed which ones of the known OA-sensitive phosphatases are expressed in *Xenopus* oocytes. Western blots revealed that the catalytic subunits of PP1, PP4, PP5, PP6 and PP2A (PP2A-C) as well as PP2A structural (PP2A-A) and regulatory (B55δ and B56ε) subunits are expressed at the same level in prophase- and metaphase II-arrested oocytes (Fig. 2a). In an attempt to identify which one corresponds to S109-phosphatase, prophase extracts were fractionated by precipitation using increasing amounts of ammonium sulfate. S109-phosphatase and PKA activities were then assayed, using as substrates pS109-GST-Arpp19 and GST-Arpp19 respectively. S109-phosphatase activity was recovered in the 40% precipitate (Fig. 2b and c) whereas PKA activity was precipitated by 60% ammonium sulfate (Fig. 2d and e). S109-phosphatase activity did no longer depend on PKI in the 40% precipitate (Fig. 2b and c), in agreement with the absence of PKA activity in this precipitate (Fig. 2d and e).

Each precipitated fraction was further analyzed by western blot with antibodies against OA-sensitive phosphatases, PKA and PKI. Consistently with the S109 phosphorylation activity measured in Fig. 2d and e, PKA was recovered in the 60% ammonium sulfate precipitate (Fig. 2f). PP2A, PP4 and PP6, as well as the scaffold PP2A-A subunit, were recovered in the 40% precipitate (Fig. 2f). In contrast, PP1 and PP5 were mostly detected in the 60% precipitate (Fig. 2f). Hence, the ammonium sulfate fractionation supports our previous results showing that S109-phosphatase is counterbalanced by PKA and indicates that it corresponds neither to PP1 nor to PP5.

### Biochemical identification of S109-phosphatase as PP2A-B55α/δ

**Fig. 3:**
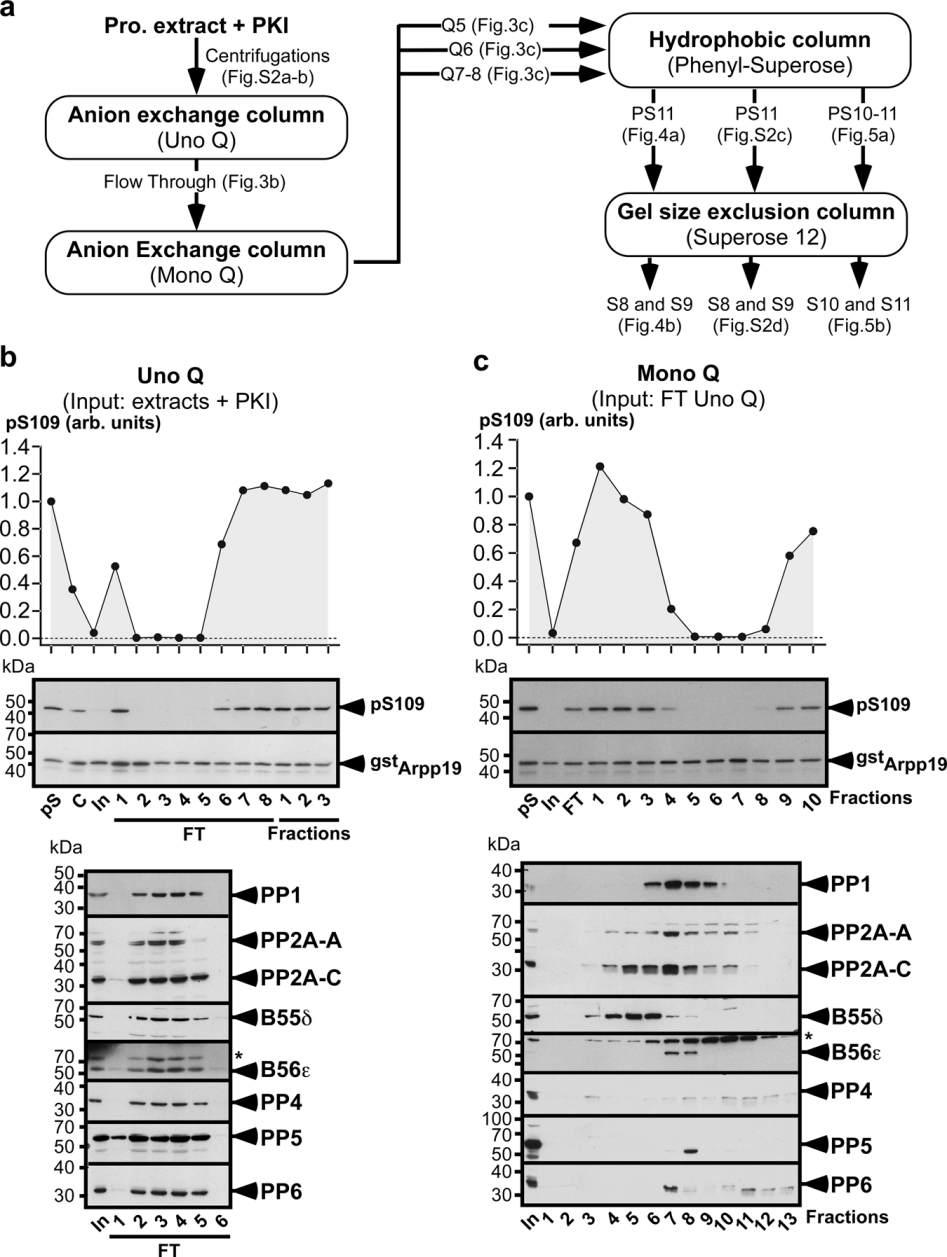
Biochemical isolation of S109-phosphatase from prophase extracts - Analysis of the output fractions of Uno Q and Mono Q columns. **a** Protocol of S109-phosphatase biochemical isolation. 20,000 prophase oocytes were lysed, centrifuged and fractionated by 4 successive steps of chromatography: two anion exchange columns (Uno Q and Mono Q), one hydrophobic column (Phenyl-Superose) and one size exclusion column (Superose 12). PKI-supplemented extracts from prophase oocytes were fractionated by Uno Q (**b**) and then Mono Q (**c**). S109-phosphatase activity was assayed in each fraction using pS109-GST-Arpp19 as a substrate (pS: phosphorylated substrate). S109 phosphorylation of GST-Arpp19 (pS109) and total GST-Arpp19 (^gst^Arpp19) were analyzed by western blot using respectively phospho-S109-Arpp19 and GST antibodies. Fractions were western blotted with antibodies against the catalytic subunits of PP1, PP2A (PP2A-C), PP4, PP5 and PP6, PP2A scaffold subunit A (PP2A-A) and PP2A regulatory subunits B55δ and B56ε. *: non-specific protein recognized by the anti-B56ε antibody. “C”: control extracts before PKI addition. “In”: input sample supplemented with PKI and loaded on the column. “FT”: flow-through. arb. units: arbitrary units. **b** Uno Q. FT and elution profile (fractions 1–3) of S109-phosphatase activity after Uno Q column and western blot analysis of fractions 1 to 6 of FT. (**c**) Mono Q. Fractions 2 to 5 of the Uno Q column FT (see **b**) were pooled and loaded on the column. Elution profile of S109-phosphatase activity after Mono Q column and western blot analysis of fractions 1 to 13. kDa: kiloDalton. Source data are provided as a Source Data file.

To identify the S109-phosphatase, a chromatography-based procedure was undertaken. PKI-supplemented prophase extracts were ultracentrifuged (Supplementary Fig. 2a and b) and the supernatant was fractionated by four successive steps of chromatography (Fig. 3a): two anion exchange columns, Uno Q and Mono Q, a hydrophobic column, Phenyl-Superose, and a size-exclusion column, Superose 12. At each step, S109-phosphatase activity was assayed in the fractions using pS109-GST-Arpp19 as a substrate. Fractions were western blotted with various antibodies against phosphatases and some were subject to LC-MS/MS experiments.

Following the Uno Q column, S109-phosphatase activity was recovered in the flow-through (Fig. 3b), as well as PP2A-B55δ, PP2A-B56ε and the catalytic subunits of PP1, PP4, PP5 and PP6 (Fig. 3b). Fractions 2 to 5 in the flow-through were pooled and loaded on a Mono Q column. S109-phosphatase activity was predominantly eluted in fractions 5–8 (Fig. 3c). Fractions 5 and 6 contain PP2A-B55δ, whereas PP4, PP5 and PP6 were not detectable in these fractions (Fig. 3c, Supplementary Table 1). Some PP1 was present in fraction 6 but not in fraction 5 (Fig. 3c, Supplementary Table 1). S109-phosphatase activity was also detected in fractions 7 and 8 that contain various amounts of PP1, PP2A-B55δ, PP2A-B56ε, PP4, PP5, PP6, and PP2C (Fig. 3c and Supplementary Table 1). Hence, PP2A-B55δ is predominant in fractions 5 and 6 whereas fractions 7 and 8 are enriched in PP1, PP2A-B56ε, PP4, PP5, PP6 and PP2C, with few PP2A-B55δ. Fraction 6 corresponds to an intermediary between both groups. The procedure to isolate S109-phosphatase was pursued by separately analyzing fraction 5, fraction 6 and a pool of fractions 7 and 8 (Fig. 3a).

**Fig. 4:**
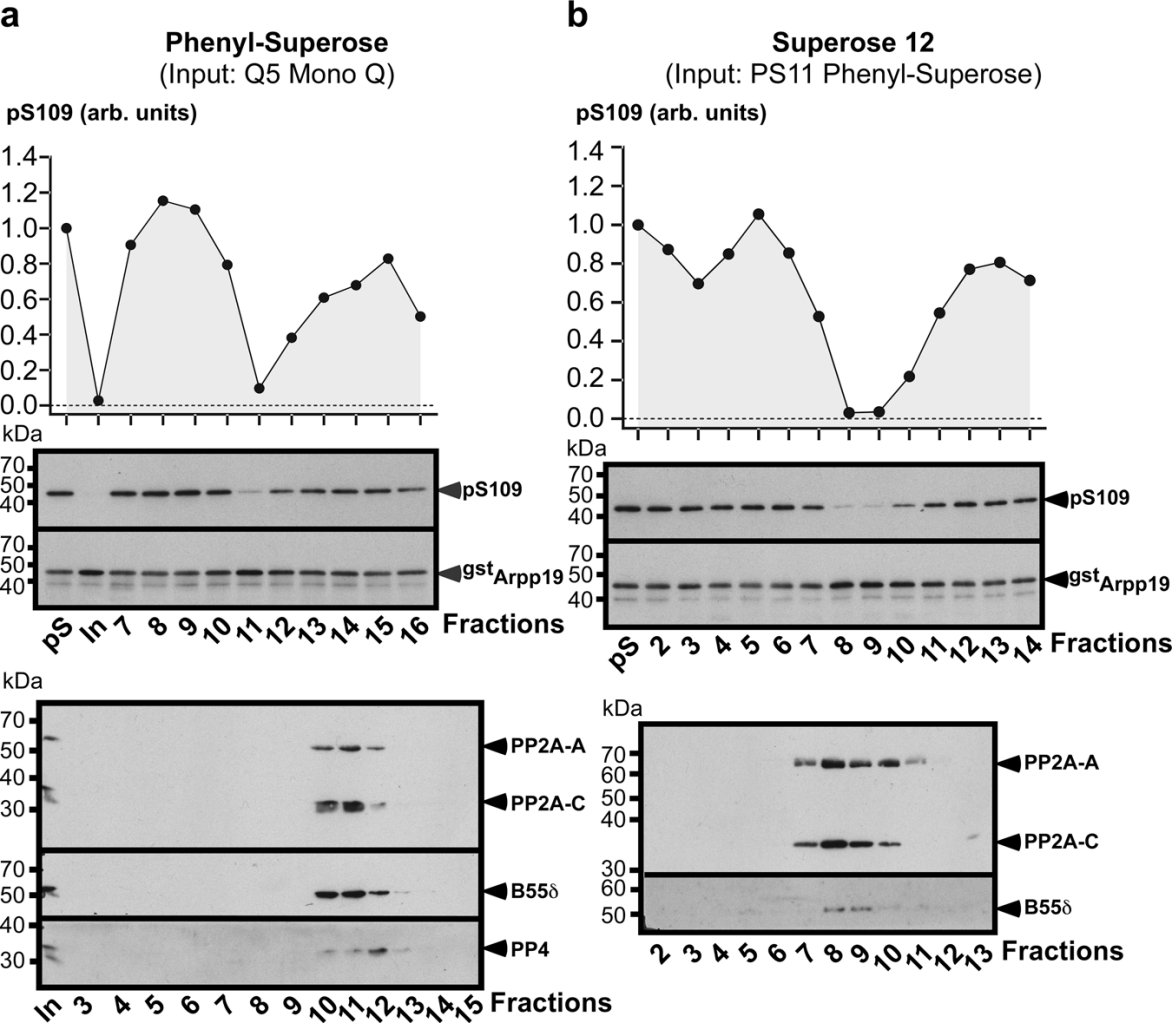
Biochemical isolation of S109-phosphatase from prophase extracts—Separation of fraction 5 from the Mono Q column with Phenyl-Superose and Superose 12 columns. Continuation of experiment illustrated in Fig. 3. “pS”: phosphorylated pS109-GST-Arpp19 substrate. “In”: input sample loaded on the column. kDa: kiloDalton. arb. units: arbitrary units. **a** Phenyl-Superose. Fraction 5 from the Mono Q column (see Fig. 3c) was loaded on the column. Elution profile of S109-phosphatase activity after Phenyl-Superose column and western blot analysis of fractions 3–15 with antibodies directed against catalytic subunits of PP2A (PP2A-C) and PP4, PP2A scaffold subunit A (PP2A-A) and PP2A regulatory subunit B55δ. **b** Superose 12. Fraction 11 from the Phenyl-Superose column (see **a**) was loaded on the column. Elution profile of S109-phosphatase activity after Superose 12 column and western blot analysis of fractions 2–13 with antibodies directed against PP2A scaffold subunit (PP2A-A), PP2A catalytic subunit (PP2A-C) and PP2A regulatory subunit B55δ. Source data are provided as a Source Data file.

Fractions 5 and 6 were subjected to Phenyl-Superose chromatography. For both fractions, S109-phosphatase activity was eluted in a single fraction, fraction 11, where the main phosphatase present is PP2A-B55α/δ with traces of PP4 (Fig. 4a, Supplementary Fig. 2c and Supplementary Table 1). Consistently with the absence of PP1, PP5 and PP6 in the input, these three phosphatases were undetectable in fraction 11 by LC-MS/MS analysis. PP1, initially present in fraction 6 of the Mono Q, was not recovered in any fraction from 7 to 16 of the Phenyl-Superose column (Supplementary Table 1). Therefore, PP2A-B55α/δ is the major phosphatase present in the fraction 11 that contains S109-phosphatase activity. This fraction was then loaded on a Superose 12 column. The maximal activity of S109-phosphatase was recovered in fractions 8 and 9 where the only phosphatase present was PP2A-B55α/δ (Fig. 4b, Supplementary Fig. 2d and Supplementary Table 1).

**Fig. 5:**
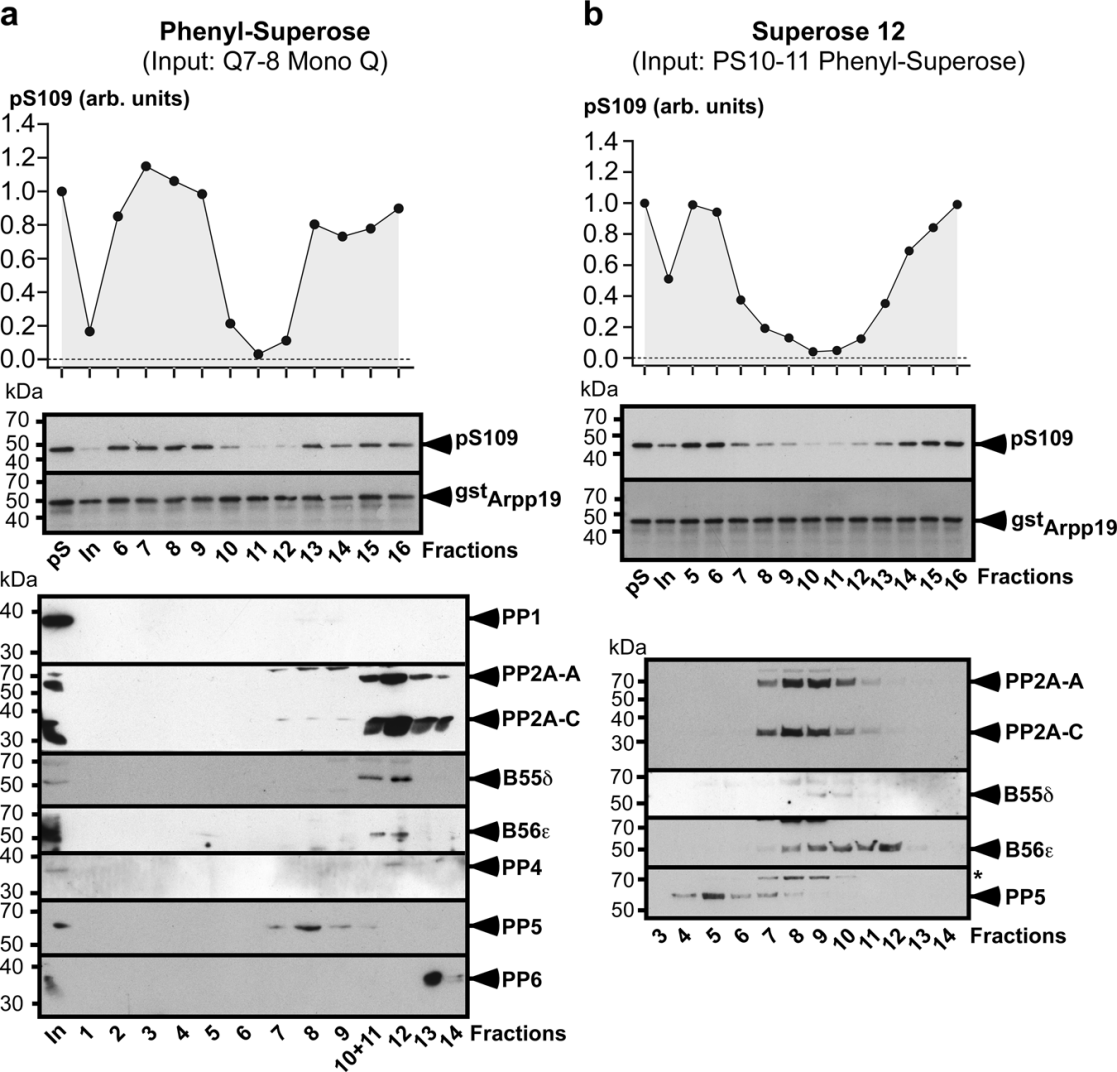
Biochemical isolation of S109-phosphatase from prophase oocyte extracts - Separation of fractions 7 and 8 from the Mono Q column with Phenyl-Superose and Superose 12 columns. Continuation of experiment illustrated in Fig. 3. “pS”: phosphorylated pS109-GST-Arpp19 substrate. “In”: input sample loaded on the column. kDa: kiloDalton. arb. units: arbitrary units. **a** Phenyl-Superose. Fractions 7 and 8 from the Mono Q column (see Fig. 3c) were pooled and loaded on the column. Elution profile of S109-phosphatase activity after Phenyl-Superose column and western blot analysis of fractions 1–14 with antibodies directed against the catalytic subunits of PP1, PP2A (PP2A-C), PP4, PP5, and PP6, PP2A scaffold subunit (PP2A-A) and PP2A regulatory subunits B55δ and B56ε. **b** Superose 12. Fraction 10 and 11 from the Phenyl-Superose column (see **a**) were pooled and loaded on the column. Elution profile of S109-phosphatase activity after Superose 12 column and western blot analysis of fractions 3–14 with antibodies directed against the catalytic subunits of PP2A (PP2A-C) and PP5, PP2A scaffold subunit (PP2A-A) and PP2A regulatory subunits B55δ and B56ε. *non-specific protein recognized by the anti-PP5 antibody. Source data are provided as a Source Data file.

Fractions 7 and 8 from the Mono Q column were pooled and subject to Phenyl-Superose chromatography. S109-phosphatase activity was eluted in fractions 10–12 (Fig. 5a). PP2A-B55δ, PP2A-B56α/β/ε and PP2C were recovered in these fractions while PP1, PP4, PP5, and PP6 were not detected or were present in trace amounts (Fig. 5a and Supplementary Table 1). Fractions 10 and 11 were pooled and loaded on a gel filtration Superose 12 column. S109-phosphatase activity was recovered in fractions 7–13, with a peak in fractions 10 and 11 (Fig. 5b). The phosphatases contained in these fractions were PP2A-B55δ, PP2A-B56α/β/ε and PP2C (Fig. 5b and Supplementary Table 1).

Hence, the biochemical procedure to isolate S109-phosphatase activity led to PP2A-A/C and excluded PP1, PP4, PP5 and PP6. As PP2C is insensitive to OA^33^, this phosphatase is unlikely responsible for S109-phosphatase activity. Remarkably, S109-phosphatase activity was carried by PP2A-A/C associated with either B55α/δ or B56α/β/ε. The biochemical isolation procedure was performed 4 times, with 3 of these 4 experiments analyzed by LC-MS/MS. Experiment 1 was presented in Figs. 3 to 5, Supplementary Fig. 2c and 2d and Supplementary Table 1, while Supplementary Tables 2 and 3 summarize the two other experiments. In experiment 2, fractions 5 to 8 from the Mono Q column were pooled before Phenyl-Superose. In experiment 3, S109-phosphatase was eluted in a single fraction from the Mono Q column. The final fractions from the Superose 12 column containing S109-phosphatase activity were analyzed by LC-MS/MS analysis (Supplementary Tables 2 and 3). PP2A-B56 isoform was recovered in the final fractions of experiment 2, in which fractions 5–8 from the Mono Q column were pooled, whereas it was barely detectable in experiment 3 where S109-phosphatase was found in a single fraction after the Mono Q column. This argues for B56 contaminating S109-phosphatase fractions when the Mono Q fractions are pooled. In contrast, PP2A-B55 isoform was recovered in all experiments (Supplementary Tables 2 and 3), arguing for PP2A-B55α/δ being the main phosphatase carrying S109-phosphatase activity.

### Arpp19 is dephosphorylated at S109 by purified PP2A-B55δ but not by purified PP1

**Fig. 6:**
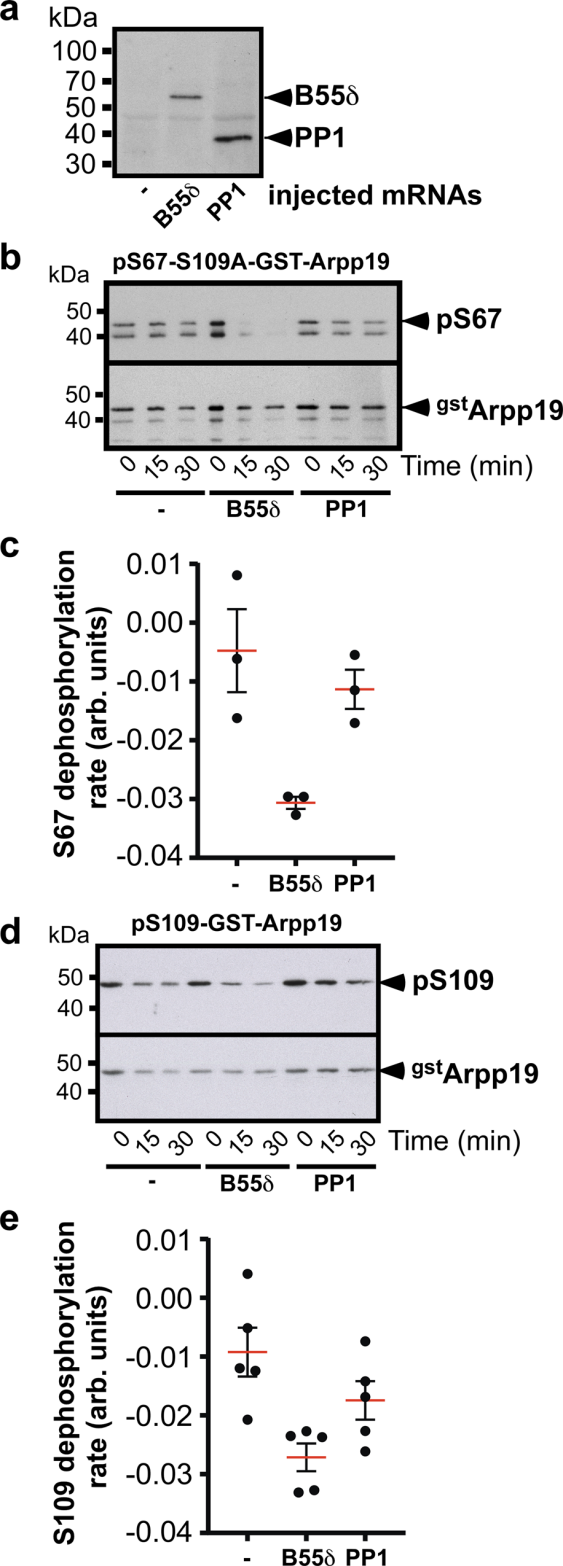
In contrast to PP1, purified PP2A-B55δ dephosphorylates Arpp19 at both S109 and S67. **a** Co-beads were incubated with extracts of prophase oocytes injected or not with mRNAs coding either His-PP1 or His-B55δ and then analyzed by western blot using an anti-Histidine antibody. The experiment was repeated 5 times with similar results. **b**–**c** S67-phosphatase activity of either PP1 or PP2A-B55δ coupled to Co-beads using pS67-S109A-GST-Arpp19 as a substrate. S67 phosphorylation of S109A-GST-Arpp19 (pS67) and total GST-S109A-Arpp19 (^gst^Arpp19) were western blotted using respectively phospho-S67-Arpp19 and GST antibodies. Time course of one representative experiment (**b**) and quantified rate of S67 dephosphorylation from 3 independent experiments (**c**). **d**–**e** S109-phosphatase activity of either PP1 or PP2A-B55δ coupled to Co-beads using pS109-GST-Arpp19 as a substrate. S109 phosphorylation of GST-Arpp19 (pS109) and total GST-Arpp19 (^gst^Arpp19) were western blotted using respectively phospho-S109-Arpp19 and GST antibodies. Time course of one representative experiment (**d**) and quantified rate of S109 dephosphorylation from 5 independent experiments (**c**). For quantifications, data are shown as mean (red bars) ± SEM. Each dot represents one experiment. kDa: kiloDalton. arb. units: arbitrary units. Source data are provided as a Source Data file.

To support the results of the biochemical procedure, we assayed Arpp19 dephosphorylation with purified enzymes. *Xenopus* catalytic His-PP1α and His-B55δ were expressed in prophase oocytes injected with their respective encoding mRNAs and affinity-purified using Co-beads (Fig. 6a). Both purified PP1 and PP2A-B55δ enzymes were used in in vitro dephosphorylation assays. PP2A-B55δ is known to dephosphorylate Arpp19 at S67^21^. We assessed that purified PP2A-B55δ was able to dephosphorylate Arpp19 at S67. A mutant form of Arpp19 that cannot be phosphorylated at S109, S109A-GST-Arpp19, was phosphorylated at S67 and used as a substrate. As expected, PP2A-B55δ efficiently dephosphorylated Arpp19 at S67 in contrast to PP1 (Fig. 6b and c). We then used pS109-GST-Arpp19 as a substrate. The reaction kinetics revealed that PP2A-B55δ efficiently dephosphorylates Arpp19 at S109 in contrast to PP1 whose rate of S109 dephosphorylation is very slow (Fig. 6d and e). Therefore, Arpp19 dephosphorylation observed in extracts or chromatography fractions can be recapitulated with purified enzymes.

### PP2A-B55δ dephosphorylates Arpp19 at S109 in prophase extracts

**Fig. 7:**
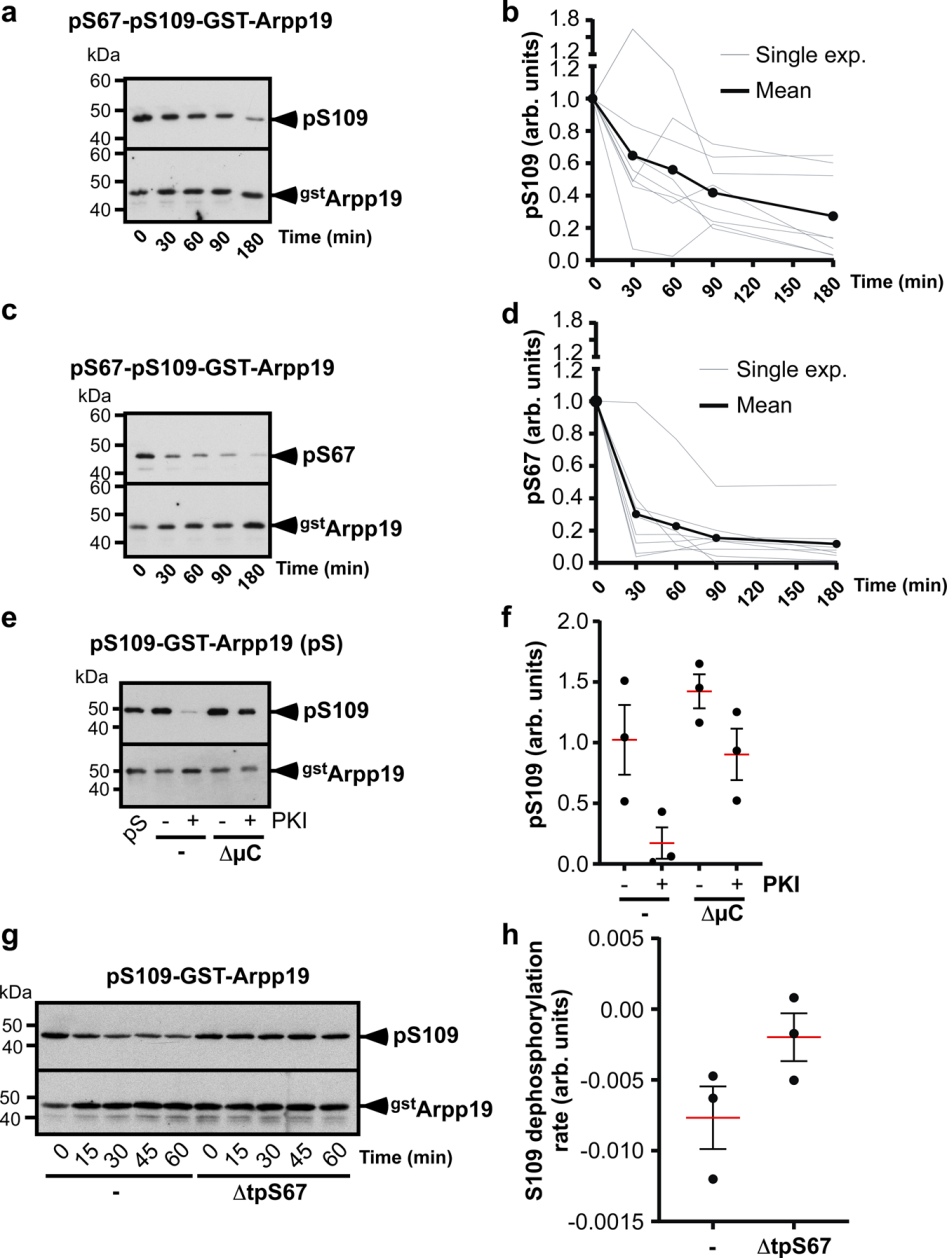
Depletion of PP2A-B55δ impairs Arpp19 dephosphorylation at S109 in prophase extracts. Prophase extracts were incubated with GST-Arpp19 phosphorylated at both S67 and S109 (pS67-pS109-GST-Arpp19) in the absence of ATP. S109-phosphatase (**a**–**b**) and S67-phosphatase activities (**c**–**d**) were assayed by western blot using phospho-S109-Arpp19, phospho-S67-Arpp19 and GST antibodies. **a**, **c** Representative time course experiments. **b, d** Quantifications of 8 time-course independent experiments. Each experiment is represented by a gray curve (Single exp.), and the mean of the 8 replicates by the black curve. arb. units: arbitrary units. **e**–**f** Prophase extracts were incubated or not with microcystin-beads (μC). After beads removal, control (–) or microcystin-depleted prophase extracts (ΔμC) were supplemented or not with PKI and assayed for S109-phosphatase activity using pS109-GST-Arpp19 as a substrate (pS: phosphorylated substrate). S109 phosphorylation of GST-Arpp19 (pS109) and total GST-Arpp19 (^gst^Arpp19) were western blotted using respectively phospho-S109-Arpp19 and GST antibodies. One representative experiment (**e**) and quantifications of S109 phosphorylation from 3 independent experiments (**f**). **g**–**h** Prophase extracts were incubated with GSH-beads coupled or not with GST-Arpp19 thiophosphorylated at S67 (tpS67) in the absence of ATP. After beads removal, control (–) or tpS67-depleted prophase extracts (ΔtpS67) were assayed for S109-phosphatase activity using pS109-GST-Arpp19 as a substrate. S109 phosphorylation of GST-Arpp19 (pS109) and total GST-Arpp19 (^gst^Arpp19) were western blotted using respectively phospho-S109-Arpp19 and GST antibodies. One representative time-course experiment (**g**) and quantified rate of S109 dephosphorylation from 3 independent experiments (**h**). For quantifications in (**f**) and (**h**), data are shown as mean (red bars) ± SEM. Each dot represents one experiment. kDa: kiloDalton. arb. units: arbitrary units. Source data are provided as a Source Data file.

If PP2A-B55δ corresponds to the S109-phosphatase, it should be active in prophase-arrested oocytes. Since PP2A-B55δ is known to dephosphorylate Arpp19 at S67^21^, its activity was assayed in prophase extracts with Arpp19 phosphorylated at both S67 and S109 (pS67-pS109-GST-Arpp19). This substrate allows monitoring the concomitant dephosphorylation of S109 and S67, respectively catalyzed by S109-phosphatase and PP2A-B55δ. As expected, S109-phosphatase dephosphorylated pS67-pS109-GST-Arpp19 at S109 (Fig. 7a and b) in a PKI- and OA-dependent manner (Supplementary Fig. 3a). Interestingly, pS67-pS109-GST-Arpp19 was also dephosphorylated at S67, strongly arguing for PP2A-B55δ being active in prophase extracts (Fig. 7c and d). This event was prevented by OA but was insensitive to PKI (Supplementary Fig. 3b), an expected result as S67 is targeted by Gwl but not by PKA^24^. Since S67-phosphorylated Arpp19 inhibits PP2A-B55δ activity, it should impair the dephosphorylation of S109^13,14^. Indeed, the kinetic parameters show that Arpp19 is not dephosphorylated at S109 as long as S67 remains phosphorylated (Fig. 7a–d). Since Greatwall is not active in prophase extracts, Arpp19 cannot be rephosphorylated at S67, accounting for the loss of its inhibitory effect toward PP2A-B55δ that can eventually dephosphorylate S109. Altogether, our results indicate that PP2A-B55δ is active in prophase oocytes and not affected by PKA.

To ascertain that PP2A-B55δ is responsible for Arpp19 dephosphorylation at S109, this phosphatase was specifically depleted from prophase extracts using its specific inhibitor, S67-phosphorylated Arpp19, coupled to GSH-beads. To avoid S67 dephosphorylation, GST-Arpp19 was thiophosphorylated with ATP-γS (tpS67-GST-Arpp19)^24^. As a positive control, microcystin-beads known to inhibit all PP2A-A/C isoforms by direct binding were used^34^. After incubation with either tpS67-GST-Arpp19-beads or microcystin-beads, supernatants and beads were separated. As expected from the pan-specificity of microcystin, PP2A subunits A, C, B56ε, and B55δ were recovered on the microcystin-beads (Supplementary Fig. 3c). In contrast, given the specificity of tpS67-GST-Arpp19, B55δ was strongly enriched in tpS67-GST-Arpp19-beads, whereas B56ε was barely detectable (Supplementary Fig. 3c). S109-phosphatase activity was then assayed in the supernatants. In the absence of PKI, Arpp19 phosphorylation at S109 was increased in PP2A-depleted supernatants with microcystin-beads (Fig. 7e and f), showing that PKA actively phosphorylates Arpp19 at S109 in the absence of PP2A. Inhibiting PKA with PKI led to a strong S109 dephosphorylation of Arpp19 in prophase extracts (Fig. 7e and f). In contrast, such a dephosphorylation was no longer observed in the microcystin-depleted supernatants (Fig. 7e and f). Importantly, the specific depletion of PP2A-B55δ with tpS67-GST-Arpp19-beads similarly abolished Arpp19 dephosphorylation at S109 (Fig. 7g and h). Altogether, these results indicate that PP2A-B55δ behaves as the S109-phosphatase in prophase extracts: it is active in dephosphorylating Arpp19 and counterbalanced by PKA.

### PP2A-B55δ dephosphorylates Arpp19 at S109 in intact oocytes

**Fig. 8:**
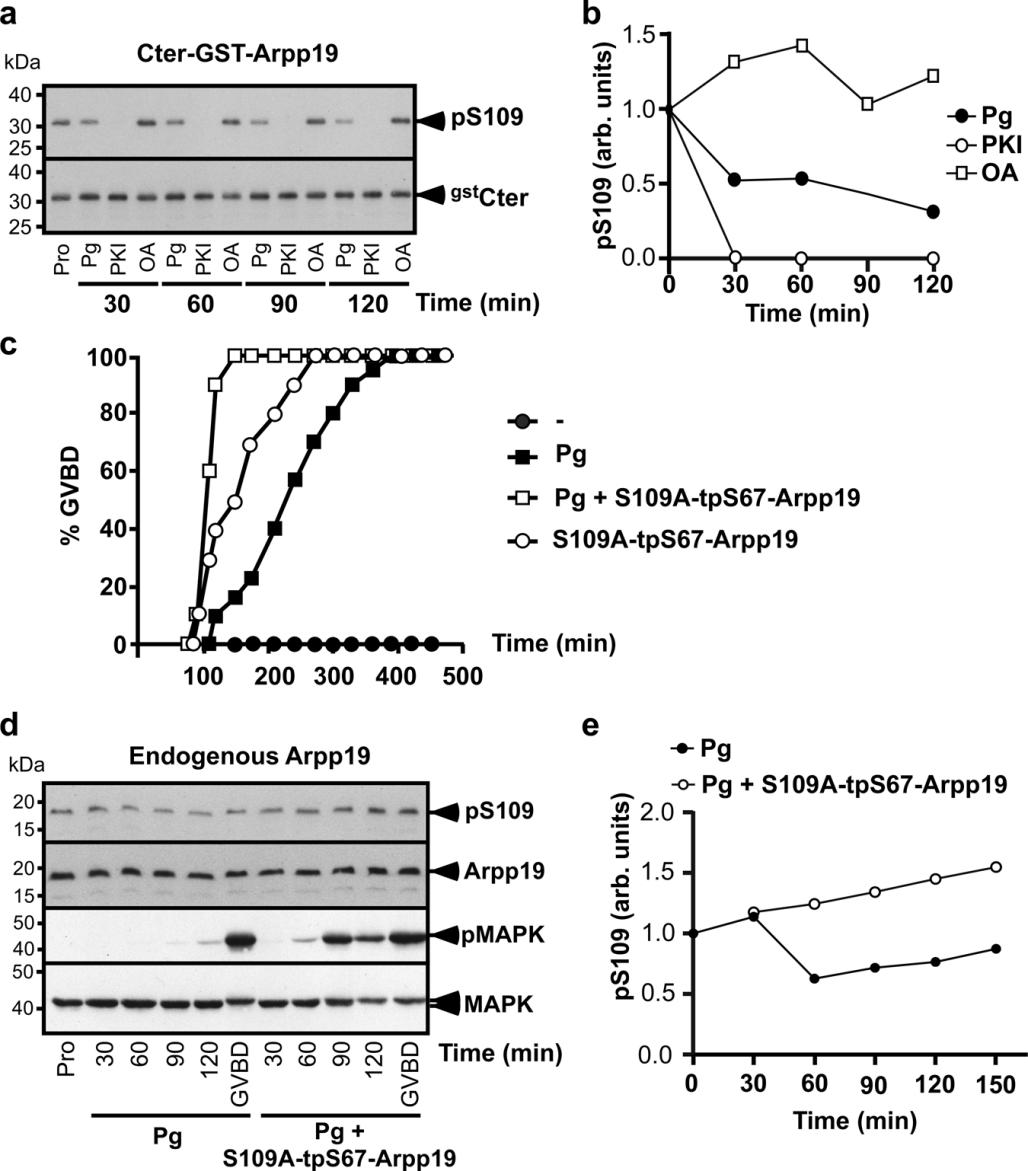
Inhibition of PP2A-B55δ prevents Arpp19 dephosphorylation at S109 in intact oocytes. **a** Prophase oocytes (Pro) were injected with Cter-GST-Arpp19 (^gst^Cter) and then stimulated with progesterone (Pg) or injected with either PKI or okadaic acid (OA) (time zero). Oocytes were collected at the indicated times and Cter-GST-Arpp19 was isolated by pull-down. S109 phosphorylation of Cter-GST-Arpp19 (pS109) and total Cter-GST-Arpp19 (^gst^Cter) were western blotted using respectively phospho-S109-Arpp19 and GST antibodies. **b** S109 phosphorylation of the experiment illustrated in (**a**) was quantified. **c**–**e** Prophase oocytes were injected or not with S109A-GST-Arpp19 thiophosphorylated at S67 (S109A-tpS67-Arpp19) and then stimulated or not with progesterone (Pg). **c** GVBD time-course. **d** Same experiment as (**c**). Oocytes were collected in prophase (Pro) or at the indicated times after Pg addition and western blotted for S109-phosphorylated endogenous Arpp19 (pS109), total endogenous Arpp19, phosphorylated MAPK (pMAPK) and total MAPK. (**e**) Quantification of S109 phosphorylation of endogenous Arpp19 from the experiment illustrated in (**d**). kDa: kiloDalton. arb. units: arbitrary units. Source data are provided as a Source Data file.

We first ascertained whether Arpp19 phosphorylation at S109 is also subject to a turnover in intact oocytes. We used as S109-phosphatase substrate a GST-truncated form of Arpp19, corresponding to its C-terminal part (68-117) (Cter-GST-Arpp19). This peptide contains S109 but lacks S67 that is phosphorylated by Gwl during meiosis resumption^24^. In vitro, Cter-GST-Arpp19 was efficiently phosphorylated at S109 by PKA and efficiently dephosphorylated at S109 when incubated in PKI-supplemented prophase extracts (Supplementary Fig. 4a and 4b). Moreover, unlike full-length Arpp19 protein^3,24^, Cter-GST-Arpp19 did not interfere with meiotic maturation (Supplementary Fig. 4c and 4d). Prophase oocytes were injected with Cter-GST-Arpp19 and either stimulated with progesterone or injected with PKI or OA. Cter-GST-Arpp19 was then isolated by pull-down at various times before Cdk1 activation. As shown in Fig. 8a and b, Cter-GST-Arpp19 was in vivo phosphorylated at S109 in prophase oocytes and was partly dephosphorylated in response to progesterone, thus reproducing the dephosphorylation of endogenous Arpp19 observed upon hormonal stimulation^3^. In the absence of progesterone, PKI injection induced the full dephosphorylation of Cter-GST-Arpp19 at S109 while OA injection increased the S109 phosphorylation level (Fig. 8a and b). Hence, S109-phosphatase is an OA-sensitive phosphatase that is active in prophase oocytes, the phosphorylation level of Arpp19 at S109 resulting from a balance between PKA and its opposing phosphatase, in favor of PKA.

Whether PP2A-B55δ also dephosphorylates Arpp19 at S109 in intact oocytes was ascertained using its specific inhibitor, tpS67-GST-Arpp19. To avoid any side-specific effect due to S109 phosphorylation, a non-phosphorylatable Arpp19 mutant, in which S109 was replaced by alanine, was used (S109A-tpS67-GST-Arpp19)^26^. S109A-tpS67-GST-Arpp19 was injected in prophase oocytes. As expected^8,24,35^, this protein promoted meiosis resumption independently of progesterone (Fig. 8c). Cdk1 activation was ascertained by western blotting phosphorylated MAPK (Fig. 8d), a marker of Cdk1 activation^36^. Arpp19 dephosphorylation at S109 was analyzed by western blot (Fig. 8d and e). As previously shown^3^, the protein was partially dephosphorylated at S109 within 1 h upon progesterone addition and was then rephosphorylated at this residue before GVBD in control oocytes (Fig. 8d and e). In contrast, Arpp19 was not dephosphorylated at S109 when PP2A-B55δ was specifically inhibited by S109A-tpS67-GST-Arpp19 (Fig. 8d and e). Hence, inhibiting PP2A-B55δ in oocytes is sufficient to abolish Arpp19 dephosphorylation at S109 induced by progesterone. PP2A-B55δ is therefore responsible for Arpp19 dephosphorylation during oocyte meiosis resumption.

### Arpp19 dephosphorylation at S109 by PP2A-B55δ is independent of Cdk1

**Fig. 9:**
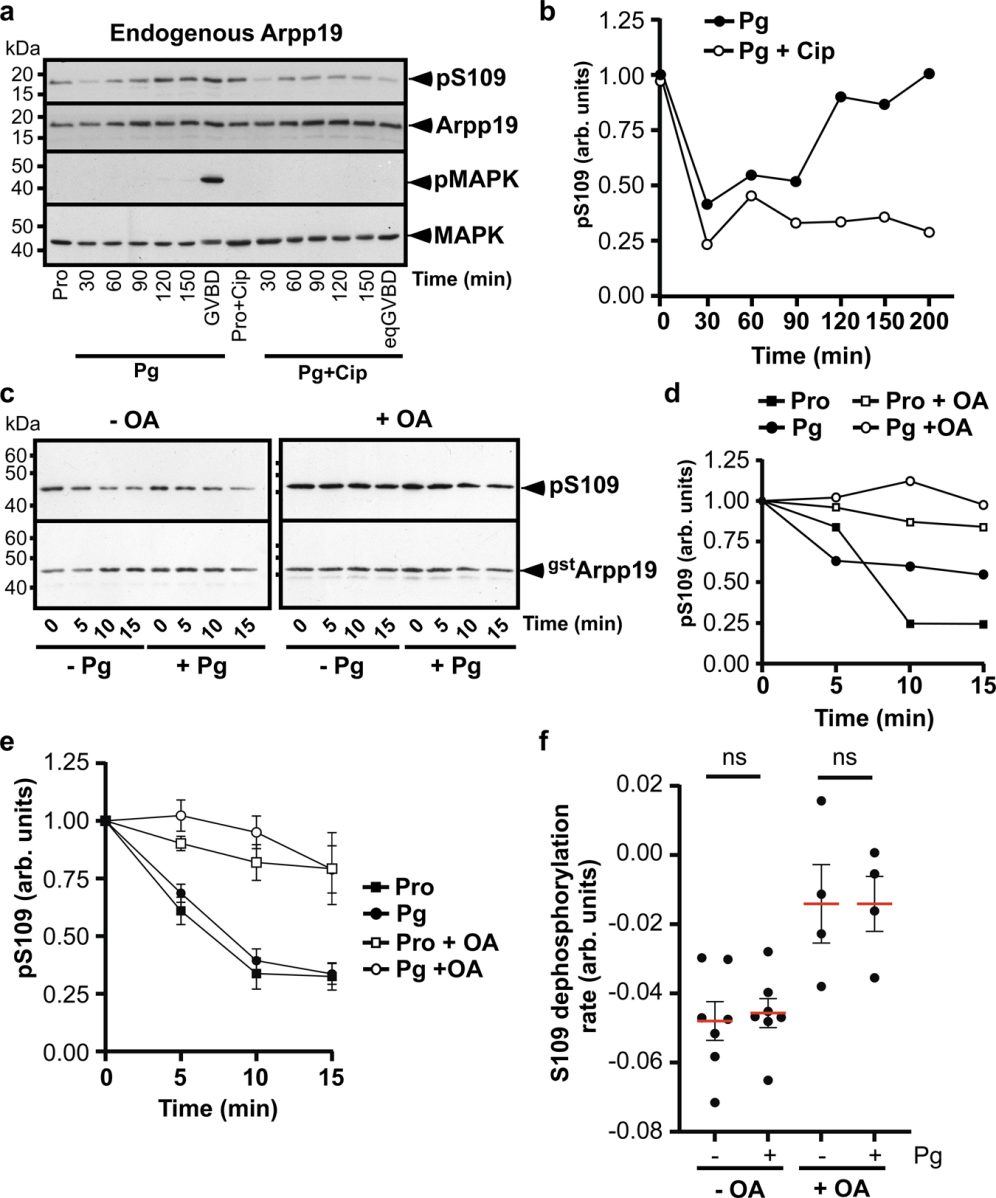
Level of PP2A-B55δ activity in intact oocytes. **a** Prophase oocytes (Pro) were injected or not with p21^Cip1^ (Cip) and then stimulated with progesterone (Pg). GVBD occurred at 200 min in control oocytes and was prevented by Cip injection. Oocytes were collected at the indicated times after Pg addition and western blotted with antibodies against S109-phosphorylated Arpp19 (pS109), total Arpp19, phosphorylated MAPK (pMAPK) and total MAPK. **b** S109 phosphorylation of the experiment illustrated in (**a**) was quantified. **c** Extracts from either prophase oocytes (–Pg) or oocytes stimulated for 1 h with progesterone (+Pg) were supplemented or not with 10 μM okadaic acid (OA) in the absence of ATP. The activity of S109-phosphatase was assayed at the indicated times, using pS109-GST-Arpp19 as a substrate. S109 phosphorylation of GST-Arpp19 (pS109) and total GST-Arpp19 (^gst^Arpp19) were western blotted using respectively phospho-S109-Arpp19 and GST antibodies. **d** Quantification of S109 phosphorylation of GST-Arpp19 from the representative experiment illustrated in (**c**). **e** Quantification of S109 phosphorylation of GST-Arpp19 from 7 independent time-course experiments. S109-phosphatase activity was assayed and quantified as in (**c**–**d**). Four experiments were performed with or without OA and 3 experiments without OA. For each condition, data are shown as mean (red bars) ± SEM. **f** Quantified rates of S109 dephosphorylation from the 7 independent experiments, described in (**e**). Four experiments were performed with or without OA and 3 experiments without OA. Data are shown as mean (red bars) ±  SEM. Each dot represents one experiment. *P* values were obtained by a two-tailed paired Student *t* test. *P* > 0.05: non-significant (ns). kDa: kiloDalton. arb. units: arbitrary units. Source data are provided as a Source Data file.

Cdk1 activation depends on PP2A-B55δ inhibition thanks to Arpp19 phosphorylation at S67 that occurs at GVBD^24^. This negative action of PP2A-B55δ is controlled by Cdk1 itself, which indirectly activates Gwl, constituting a positive feedback loop^5^. Remarkably, our present results reveal that PP2A-B55δ is the phosphatase that dephosphorylates Arpp19 at S109 upstream Cdk1 activation, as early as 1 h after progesterone stimulation (Fig. 8d and e), a necessary event to promote meiotic resumption^3^. Hence, both steps, critical for Cdk1 activation, involve PP2A-B55δ and Arpp19 but occur sequentially over time. To ensure that the early action of PP2A-B55δ is disconnected from its late function, we investigated whether Arpp19 dephosphorylation at S109 is independent of Cdk1 activity. Prophase oocytes were injected with a specific inhibitor of Cdk1^37^, the protein p21^Cip1^ and were then stimulated with progesterone. In control oocytes, Arpp19 was phosphorylated at S109 in prophase, partially dephosphorylated at S109 within 1 h after progesterone addition and then rephosphorylated at this residue just before GVBD (Fig. 9a). P21^Cip1^ injection efficiently prevented Cdk1 activation in response to progesterone (Fig. 9a). Under these conditions, Arpp19 was still dephosphorylated at S109 in response to progesterone and was then maintained in its partially dephosphorylated state (Fig. 9a and b). This result indicates that PP2A-B55δ actively dephosphorylates Arpp19 at S109 well before and independently of Cdk1 activation. Both actions of PP2A-B55δ are therefore temporally and functionally disconnected in the oocyte.

### PP2A-B55δ activity is not regulated in the early stages of meiosis resumption

In prophase oocytes, Arpp19 phosphorylation at S109 depends on a balance between PKA and PP2A-B55δ in favor of PKA. Arpp19 dephosphorylation could be either promoted by the sole inhibition of PKA or by the concomitant up-regulation of PP2A-B55δ activity. To address this issue, we assayed PP2A-B55δ activity in extracts from oocytes either in prophase or collected 1 h after progesterone stimulation. As expected, endogenous Arpp19 was partially dephosphorylated at S109 at that time (Supplementary Fig. 5). PP2A-B55δ substrate, pS109-GST-Arpp19, was added to oocyte lysates in the presence or in the absence of OA, and its dephosphorylation was ascertained as a function of time. As shown in Fig. 9c and d, Arpp19 was dephosphorylated in an OA-dependent manner, at a similar rate in extracts from oocytes in prophase or stimulated by progesterone. The experiment was performed 7 times using different females (Fig. 9e and f). No statistical difference was observed regarding the S109 dephosphorylation level nor the rates of dephosphorylation by PP2A-B55δ at both stages. Therefore, PP2A-B55δ activity is not up-regulated by progesterone at the early stages of meiosis resumption and Arpp19 dephosphorylation at S109 solely depends on PKA downregulation.

## Discussion

In all vertebrates, a high level of PKA activity arrests oocytes in prophase of the first meiotic division for long periods. In *Xenopus*, Arpp19 phosphorylated at S109 by PKA plays an important part in maintaining the prophase block^3^. Progesterone releases this block by decreasing cAMP levels and PKA activity within 1 h, resulting in a partial dephosphorylation of Arpp19 at S109 sufficient to trigger a signaling pathway that leads, 3–5 h later, to Cdk1 activation^3,38–40^. We have now uncovered the phosphatase responsible for Arpp19 dephosphorylation at S109, unexpectedly finding it to be the same PP2A isoform that opposes Cdk1 during M-phase entry.

PP1 was initially an attractive candidate to oppose the action of PKA during oocyte prophase arrest. PKA and PP1 antagonistically regulate the activity of many physiological effectors involved in cell cycle regulation. In particular, PP1 inhibition blocks progesterone and PKI-induced meiotic maturation in *Xenopus* oocytes. PP1 was thus proposed to dephosphorylate a PKA substrate, still unknown at that time^41^. PP1 also positively regulates mitosis by contributing to Cdc25C activation and by controlling structural events such as centrosome splitting, spindle assembly and microtubules-kinetochores attachment^42^. PP1 activity is then repressed at mid-mitosis by Cdk1 phosphorylation and reactivated at the end of mitosis where it contributes to the dephosphorylation of Cdk1 substrates^42–44^. Nevertheless, we have ruled out the possibility that PP1 is responsible for Arpp19 dephosphorylation on S109. PP1 did not associate with S109-phosphatase activity in the ammonium sulfate precipitates nor after the Mono Q column and the purified enzyme was not efficient in dephosphorylating Arpp19 at S109 in vitro.

All our in vitro or in vivo approaches demonstrate that the phosphatase that acts on Arpp19 at S109 is a PP2A isoform. We were further able to determine that this activity is predominantly carried by the B55 subunit, i.e., the same isoform that participates as an essential component of the M-phase switch. We detected B55, in some cases accompanied by B56, in the active S109-phosphatase fractions obtained from all three biochemical isolation procedures analyzed by western blot and LC-MS/MS, as well as in an initial isolation experiment monitored by western blot only. While PP2A-B55 is thus clearly the main phosphatase responsible for S109 dephosphorylation of Arpp19, PP2A-B56 can also potentially contribute, at least in extracts. In contrast, inhibiting specifically PP2A-B55δ using S67-phosphorylated Arpp19 in intact oocytes fully abolishes S109 dephosphorylation of endogenous Arpp19 induced by progesterone and even increases its phosphorylation level. These experiments establish that in vivo, PP2A-B55 is the physiological phosphatase that dephosphorylates Arpp19 at S109. The minor contribution of PP2A-B56 in oocyte extracts could result from its release from discrete subcellular compartments during extract preparation. It has been established that PP2A-B56 localizes specifically to kinetochores and centromeres, participating in chromatid-microtubule interactions and silencing of the spindle assembly checkpoint^45,46^. Likely relating to this M-phase role, PP2A-B56 is involved in the metaphase II-arrest of *Xenopus* oocyte by regulating the APC/C inhibitor, XErp1^47,48^. These M-phase roles of PP2A-B56 are not directly connected to Cdk1 regulation and do not involve Arpp19. Accordingly, Arpp19 lacks the two known binding motifs for PP2A-B56 but includes bipartite recognition determinants for PP2A-B55^49–51^.

Each of the four B subfamilies comprises several isoforms with very closely related sequences, no discernible differences in their substrate binding pockets and substantial substrate specificity overlap^52^. In the present study, our biochemical isolation procedures identify B55δ as being associated with PP2A-A/C that dephosphorylates Arpp19 at S109. B55α was also detected by LC-MS/MS experiment. Since we have no specific anti-B55α antibody, we cannot exclude that S109-phosphatase activity is a mixture of PP2A-B55α and PP2A-B55δ. Nevertheless, in *Xenopus* oocytes, B55δ accounts for 70% or more of the total B55 subunits, with B55α and B55β antibodies failing to detect endogenous proteins, due to their low expression level^8,53^. Moreover, Arpp19 is not detected within the protein interactome of PP2A-B55α^54^. We therefore ascribe the majority of the phosphatase responsible for dephosphorylating Arpp19 at S109 to the PP2A subset containing the B55δ subunit. Similarly, in *Xenopus* egg extracts, PP2A-B55δ is the key phosphatase isoform that acts on Cdk1 substrates, a property not shared by the other *Xenopus* PP2A-B55 holoenzymes^8,53^ and is inhibited by Arpp19 phosphorylated at S67^13^.

Our results revealed that Arpp19 phosphorylation at S109 is subject to dynamic turnover in prophase-arrested oocytes. The two mutually antagonistic enzymes, PKA and PP2A-B55δ, work simultaneously, with the action of PP2A-B55δ being swamped by PKA. This contrasts with other systems in which PKA plays a critical role in phosphate removal by regulating protein phosphatase activities^55^, for example by phosphorylating B56 and activating PP2A-B56δ in human brain^56^. The simultaneous activities of PKA and PP2A-B55δ result in a futile phosphorylation cycle. This not only renders impossible a full switch-like interconversion of Arpp19 phosphorylation state, but also allows the two opposed active enzymes to carry important functions independently of each other. As such, PKA certainly targets substrates other than Arpp19, important to keep oocytes arrested in prophase, such as Cdc25^57^ or, similarly to Wee1b in mouse^58^, Myt1. Meantime, the sustained activity of PP2A-B55δ also ensures the stability of the prophase arrest by impeding Cdk1 activation and its substrates phosphorylation.

Remarkably, both Arpp19 sites important for the control of meiosis resumption, S109 and S67, are dephosphorylated by a unique phosphatase, PP2A-B55δ. The function of Arpp19 phosphorylation at S67 in converting this protein into a PP2A-B55δ inhibitor is well documented, whereas the action of S109 phosphorylation of Arpp19 in ensuring the prophase arrest remains unknown. It is tempting to speculate that, by mirroring the function of S67-phosphorylated Arpp19, S109-phosphorylated Arpp19 regulates one of its regulatory kinases or phosphatases. Such a dual function has been described for two Arpp19-related proteins, DARPP-32 and Arpp16. Depending on its phosphorylation by PKA or by Cdk5 at two distinct sites, DARPP-32 acts as an inhibitor of either PP1 or PKA^59,60^. Arpp16, exclusively expressed in the brain, and Arpp19, ubiquitously expressed, are alternatively spliced variants of the same gene^61,62^. Like Arpp19, Arpp16 is phosphorylated by Gwl at S46 and by PKA at S88^61,63^. As reported for Arpp19, Arpp16 is converted into a strong inhibitor of specific PP2A isoforms when phosphorylated by Gwl^63^. Interestingly, the PKA phosphorylated form of Arpp16 at S88 makes PP2A non-inhibitable^64^ and, in contrast to Arpp19, phosphorylation of Arpp16 at S88 and S46 is mutually antagonistic^26,64^. These findings raise the question of whether Arpp19 is a dual-function protein, like DARPP-32 or Arpp16. Although our preliminary attempts failed to show that S109-phosphorylated Arpp19 interacts with PP2A and regulates its activity, this hypothesis deserves deeper investigation. Another possibility is that Arpp19 behaves as a PP1 inhibitor when phosphorylated by PKA, similarly to DARPP-32. In response to progesterone, S109 dephosphorylation of Arpp19 by PP2A-B55δ would activate PP1, an event that positively regulates entry into cell division^41,65^. Although the catalytic subunit of PP1 is not regulated in vitro by S109-phosphorylated Arpp19^13^, this does not exclude the possibility that Arpp19 could control in vivo PP1 through its regulatory subunits and with respect to specific physiological substrates.

**Fig. 10:**
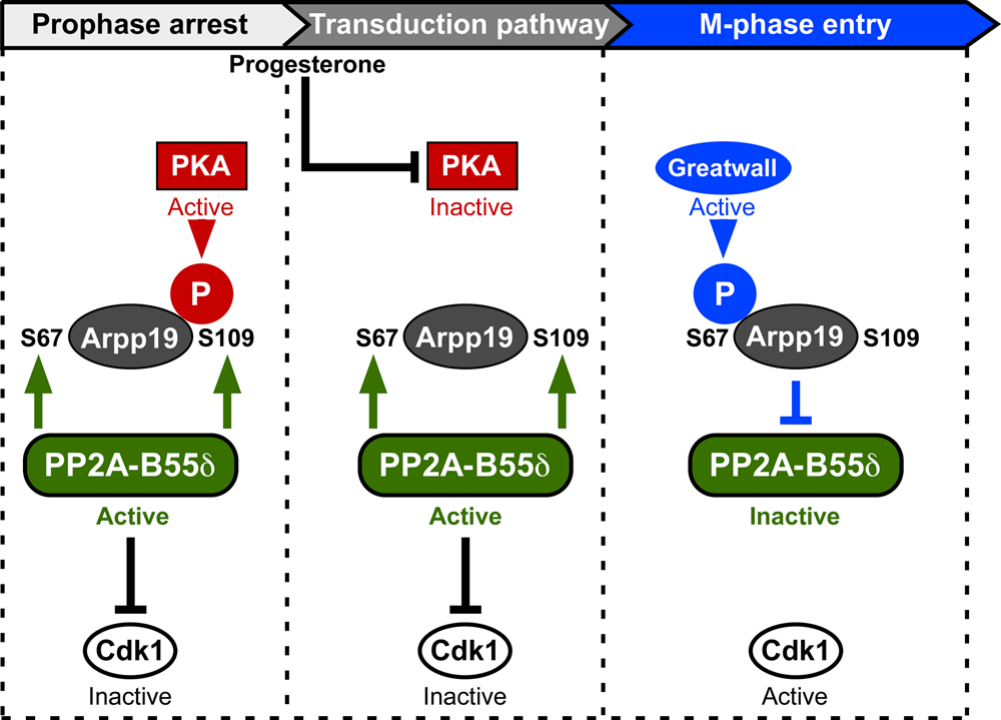
A reciprocal regulation of PP2A-B55δ and Arpp19 orchestrates the timing of the first meiotic division. Prophase arrest (left box): both PP2A-B55δ and PKA are active, resulting in Arpp19 phosphorylation at S109. S109-phosphorylated Arpp19 locks the oocyte in prophase by an unknown mechanism, PKA contributes to this arrest through Arpp19 phosphorylation and possibly other substrates, PP2A-B55δ prevents Cdk1 activation. Transduction pathway (middle box): in response to progesterone, PKA is inhibited while PP2A-B55δ stays active, allowing Arpp19 dephosphorylation. Dephosphorylated Arpp19, possibly together with other dephosphorylated PKA substrates, launches a several hours long transduction pathway. Active PP2A-B55δ prevents Cdk1 activation, generating the time window necessary to set up the cascade of molecular events required for Cdk1 activation. M-phase entry (right box): Gwl is activated by a Cdk1 activity threshold and phosphorylates Arpp19 at S67 Hence, PP2A-B55δ is inhibited and Cdk1 fully activated. This hysteretic switch triggers M-phase entry.

Our results highlight how the reciprocal regulation of PP2A-B55δ and Arpp19 orchestrates the timing of the first meiotic division in *Xenopus* oocytes in a 3-step process (Fig. 10). First, S109-phosphorylated Arpp19 and PP2A-B55δ secure the prophase arrest: PP2A-B55δ prevents any protein phosphorylation that could lead to unscheduled Cdk1 activation, while the action of S109-phosphorylated Arpp19 is not yet known. Second, progesterone triggers PKA inhibition while PP2A-B55δ stays active. As a consequence, Arpp19 is dephosphorylated at S109 and launches a several hours signaling pathway that ends with Cdk1 activation. Keeping PP2A-B55δ active prevents premature activation of Cdk1 and thus allows sufficient time for the signaling pathway, especially the translation of new proteins required for meiotic divisions. The third step is a switch conversion, relying on a threshold of a few active Cdk1 molecules. These activate Gwl, which in turn phosphorylates Arpp19 at S67 and inhibits PP2A-B55δ, setting up the bistable switches that govern irreversible M-phase entry^11,28^. Since PP2A-B55δ is inhibited, Arpp19 can be rephosphorylated at S109. Indeed, this is achieved by a kinase distinct from PKA and depending on Cdk1 activity^3^. The role of this new S109 phosphate, if any, is unknown. Hence, we discovered that a double function of PP2A-B55δ and its substrate, Arpp19, governs oocyte meiosis resumption, opening new avenues in the control of oocyte meiosis, but more widely in the control of cell cycle progression and its dysfunction in human pathologies related to fertility and cancer.

## Methods

### Materials

*Xenopus laevis* adult females (Centre de Ressources Biologiques Xenopes, CNRS, France) were bred and maintained according to current French guidelines in the conventional IBPS aquatic animal facility, with authorization: Animal Facility Agreement: #A75-05-25. All experiments were subject to ethical review and approved by the French Ministry of Higher Education and Research (reference APAFIS#14127-2018031614373133v2).

All reagents, unless otherwise specified, were from Sigma. Okadaic acid (OA), magnetic GSH-beads and Co-beads were purchased from Enzo Life Sciences, Promega and Clontech Laboratories respectively. Uno Q-25 was purchased from Bio-Rad, Mono Q 4.6/100PE, Phenyl-Superose HR5/5 and Superose 12 HR10/30 were from GE Healthcare.

### *Xenopus* Oocyte handling

Full-grown prophase oocytes were obtained from unprimed *Xenopus laevis* females as described^66^. Oocytes were microinjected with OA (2 μM), His-K71M-Gwl mRNA (150 μg), His-catalytic PP1 mRNA (30 ng), His-B55δ mRNA (30 ng) or the following recombinant proteins: Cter-GST-Arpp19 (125 ng, 250 ng, or 500 ng), tpS67-S109A-Arpp19 (250 ng), p21^Cip1^ (35 ng) or PKI (75 ng) in 50 nl final volume per oocyte. A 2 μM progesterone was added to the external medium. Each in vivo experimental condition was applied to groups of 15–30 oocytes. Oocytes were referred to as GVBD when the first pigment rearrangement was detected at the animal pole.

### Western blots

For all proteins except endogenous Arpp19, an equivalent of 0.6 oocyte was subjected to SDS gel electrophoresis (12, 10 or 8%)^67^ and then transferred onto nitrocellulose as described^68^. To visualize endogenous Arpp19 protein, an equivalent of 1.2 oocytes was loaded on 15.5% Tris-Tricine gels^69^. The antibodies directed against the following proteins were used: Arpp19^70^ (1:1,000, gift of Dr Angus Nairn, Yale University, USA), S109-phosphorylated Arpp19^3^ (1:500), S67-phosphorylated Arpp19^24^ (1:1,000), MAP kinase (1:1,000, Santa Cruz SC-154), phosphorylated MAP kinase (1:1,000, Cell Signaling 9106), PKA catalytic subunit (1:1,000, Abcam ab26322), PKI (1:1,000, Abcam ab122816), PP1 catalytic subunit (1:100, gift of Dr M. Bollen, KU Leuven, Belgium), PP2A-C catalytic subunit^71^ (1:500), PP2A-A scaffold subunit^71^ (1:500), B55δ regulatory subunit^8^ (1:500, gift of Dr S. Mochida, Kumamoto University, Japan), B56ε regulatory subunit^8^ (1:500, gift of Dr S. Mochida, Kumamoto University, Japan), PP4 catalytic subunit (1:500, Abcam ab115741), PP5 catalytic subunit^8^ (1:500, gift of Dr S. Mochida, Kumamoto University, Japan), PP6 catalytic subunit (1:500, Bethyl A300-844A), poly-His (1:1000, Sigma A-7058) and GST (1:10,000, Sigma A-7340). Appropriate horseradish peroxidase-labeled secondary antibodies (Jackson Immunoresearch) were revealed by chemiluminescence (Pierce). All western blots are representative of at least three different experiments. Full scans of all western blots are provided as a Source Data file.

### Oocyte extracts, PP2A depletion, ammonium sulfate precipitation

Metaphase II oocytes were homogenized in 10 volumes of Extraction Buffer (EB: 80 mM β-glycerophosphate pH 7.3, 20 mM EGTA, 15 mM MgCl_2_) and centrifuged at 15,000 × *g* for 30 min at 4 °C. Extracts were then supplemented with 1 mM ATP, 100 mM MgCl_2_ and 1 μM OA. Prophase oocytes were homogenized in 5 volumes of Purification Buffer (PB: 80 mM β-Glycerophosphate pH 7.4, 10 mM EDTA, 30 mM NaCl, 3 mM DTT) and centrifuged at 15,000 × *g* for 10 min at 4 °C. Extracts were then supplemented or not with 1 μM or 10 µM OA for 30 min at 18 °C, or with hexokinase (0.1 Units per µl) and 10 mM glucose, in the presence or in the absence of 20 mM phosphocreatine for 15 min at 18 °C. Recombinant PKI was added to the extracts at 0.15 mg/ml final concentration. For PP2A depletion, oocyte extracts were incubated twice for 30 min at 4 °C with either 50 μl of microcystin-Sepharose beads (Upstate) or 20 μl of magnetic GSH-beads, previously coupled or not to 2.5 µg of S67-thiophosphorylated GST-Arpp19. Supernatants were then used for further analysis after beads removal. For ammonium sulfate precipitation, proteins of extracts from 100 oocytes were precipitated by salting out using ammonium sulfate, successively 20%, 40 and 60% as described^72^. Ammonium sulfate pellets were resuspended in 1 ml of PB.

### Cloning and recombinant protein purification

Plasmids encoding either GST-tagged wild-type Arpp19^3^, GST-S109A-mutated Arpp19^3^, GST-p21^Cip1^^37^, Histidine-tagged PKA catalytic subunit (a gift from Susan Taylor, Addgene plasmid #14921) and PKI^3^ were previously described.

The cDNAs encoding either *Xenopus* catalytic PP1α and B55δ subunit were purchased from Thermo Fisher (ppp1ca 379914 Clone ID: 4682749 MXL1736-202771954) and GE healthcare (XCG ppp2cb cDNA cl5073873) and cloned into a pRN3 vector by PCR using primers encoding N-terminus Histidine-tag. The cDNA encoding the Cter-Arpp19 (amino-acids 68-117 of Arpp19) was cloned by PCR into a pGEX-6P-1 vector (See Supplementary Table 4). Recombinant proteins were produced in *E. coli* by autoinduction^73^. Wild-type, mutated and truncated GST-Arpp19 proteins, GST-p21^Cip1^, His-PKA catalytic subunit and PKI were purified as described in^3,37,74^, dialyzed overnight against Phosphate Buffered Saline (PBS: 4.3 mM KH_2_PO_4_, 1.4 mM Na_2_HPO_4_ pH 7.4, 13.7 mM NaCl, 2.7 mM KCl) and stored at −80 °C.

### In vitro phosphorylation of Arpp19 and GST pull-down

His-K71M-Gwl was obtained as described^24^. In vitro phosphorylation by PKA or thiophosphorylation by Gwl of recombinant GST-Arpp19 were respectively described in^26^ and^24^. The double S67-S109 phosphorylated form of Arpp19 (pS67-pS109-Arpp19) and S67-phosphorylated form of S109A-Arpp19 (pS67-S109A-Arpp19) were produced by incubating 1 μg of recombinant GST-Arpp19, previously coupled to 20 μl of magnetic GSH-beads, in 40 µl of metaphase II extracts for 2 h at 30 °C. After washing in PB, beads were stored at 4 °C for future use. In in vitro assays using purified phosphatases, S67-S109A-Arpp19 was phosphorylated in metaphase II extracts prior its elution from GSH-beads. For GST pull-down, GST-Arpp19 proteins were recovered from oocyte lysates as described previously^24^.

### PP1 and B55δ purification

mRNAs encoding Histidine-tagged catalytic PP1 and B55δ subunits were in vitro transcribed using mMESSAGE mMACHINE T3 Transcription Kit (Invitrogen, AM1348), in vitro polyadenylated using Poly(A) Tailing Kit (Invitrogen, AM1350) and purified using RNeasy Mini Kit (Qiagen, 74104). Prophase oocytes were injected with mRNAs, incubated for 18 h to allow mRNA expression and then homogenized in 10 volumes of Histidine Buffer (HB: 25 mM Hepes pH 7.2, 2 mM MgCl_2_, 1 mM β-glycerophosphate). After centrifugation at 10,000 xg for 15 min at 4 °C, lysates from 5 oocytes expressing His-B55δ or 7.5 oocytes expressing His-PP1 were incubated with 40 μl of Co-beads for 2 h at 4 °C. After washing in HB, PP1 and PP2A-B55δ bound to Co-beads were either western blotted or assayed for phosphatase activities.

### S109-phosphatase, S67-phosphatase and PKA assays

All assays used GST-Arpp19 or S109A-GST-Arpp19 at 1 μM final concentration.

Oocyte extracts, ammonium sulfate precipitates and fractions from chromatography columns: 1 μg of either pS109-GST-Arpp19 or GST-Arpp19, respectively used as substrates for S109-phosphatase or PKA, was coupled to 20 μl of magnetic GSH-beads for 30 min at 18 °C. After washing in PBS, GSH-beads coupled to Arpp19 proteins were incubated for 3 h at 30 °C with either 20 μl of oocyte extracts or 40 μl ammonium sulfate precipitates or 100 μl fractions from chromatography columns, supplemented with 1 mM ATP and 100 mM MgCl_2_. pS109-GST-Arpp19 or GST-Arpp19 coupled to beads were then isolated and western blotted using antibodies directed against S109-phosphorylated Arpp19 and GST. In some experiments, doubly phosphorylated pS67-pS109-GST-Arpp19 was used as a substrate, under the same conditions as those described above, and western blotted using antibodies directed against either S109- or S67-phosphorylated Arpp19 and GST.

Purified PP1 and PP2A-B55δ: PP1 and PP2A-B55δ bound to Co-beads were incubated with 60 μl of HB supplemented with 100 mM MgCl_2_ and substrates eluted from beads, either pS109-GST-Arpp19 or pS67-S109A-GST-Arpp19. At the indicated times, the reaction solution was centrifuged at 5000 × *g* for 5 s and 4 μl of supernatant were collected and analyzed by western blot using antibodies against either S109- or S67-phosphorylated Arpp19 or GST.

### Quantifications and statistics

All western blot signals were quantified using Image J software. To quantify the endogenous level of Arpp19 phosphorylation in in vivo experiments, the S109 phosphorylation signal was divided by the corresponding total endogenous Arpp19 signal (pS109/^total^Arpp19) for each condition. pS109/^total^Arpp19 ratios were then normalized to the ratio of prophase oocytes.

In assays using recombinant GST-Arpp19, S109 phosphorylation signal was divided by its corresponding GST signal (pS109/^gst^Arpp19) for each sample. In the phosphatase biochemical isolation and the time-course experiments, pS109/^gst^Arpp19 ratios were normalized to pS109/^gst^Arpp19 ratios of either the starting substrate or the time “0” respectively. For the other in vitro phosphatase assays, pS109/^gst^Arpp19 ratios were normalized to the main value of pS109/^gst^Arpp19 ratios of all biological replicates in order to compare them with each other. Each biological replicate was then plotted as an individual dot together with their mean and SEM values.

The graphs were prepared using the software Prism 8.

### Biochemical isolation of S109-phosphatase

In total, 20,000 prophase oocytes were homogenized at 4 °C in 5 volumes of PB and successively centrifuged at 4 °C at 300 × *g* for 5 min, at 1100 × *g* for 20 min, at 15,000 × *g* for 20 min and at 100,000 × *g* for 2 h (rotor TFT-45). The supernatant was then incubated with recombinant PKI at 0.15 mg/ml final concentration for 30 min at 30 °C and then loaded on a Uno Q-25 column previously equilibrated in PB. Proteins were eluted with a linear gradient of 0 to 1 M NaCl. The flow through containing S109-phosphatase activity was loaded on a Mono Q 4.6/100PE column equilibrated in PB. Proteins were eluted with a linear 0 to 0.6 M NaCl gradient. Active S109-phosphatase fractions were dialyzed overnight against Dialysis Buffer (DB: 20 mM Hepes pH 7.4, 1 mM EDTA and 1 mM DTT) and then supplied with 1 M (NH_4_)_2_SO_4_. Fractions were loaded on a Phenyl-Superose HR5/5 column equilibrated in DB supplemented with 1 M (NH_4_)_2_SO_4_. Proteins were eluted with a linear 1 to 0 M (NH_4_)_2_SO_4_ gradient. The active S109-phosphatase fractions were then lyophilized, resuspended in 200 μl of DB and further loaded on a gel size exclusion Superose 12 HR10/30 column.

### LC-MS/MS data acquisition and processing

#### Sample preparation

Proteins were digested overnight at 37 °C with trypsin (Promega) in a 25 mM NH_4_HCO_3_ buffer (0.2 μg trypsin in 20 μl). The resulting peptides were desalted using ZipTip μ-C18 Pipette Tips (Pierce Biotechnology).

#### Data acquisition

Samples were analyzed using an Orbitrap Q-Exactive Plus coupled to a Nano-LC Proxeon 1000 equipped with an easy spray ion source (Thermo Scientific). On the Q-Exactive Plus instrument, peptides were loaded with an online preconcentration method and separated by chromatography using a Pepmap-RSLC C18 column (0.75 × 500 mm, 2 μm, 100 Å, from Thermo Scientific), equilibrated at 50 °C and operated at a flow rate of 300 nl/min. Peptides were eluted by a gradient of solvent A (H_2_O, 0.1 % formic acid) and solvent B (100% acetonitrile, 0.1% formic acid). The column was first equilibrated 5 min with 95% of A, then B was raised to 35% in 93 min and finally, the column was washed with 80%B during 10 min and re-equilibrated at 95% A during 10 min. Peptides masses were analyzed in the Orbitrap cell in full ion scan mode at a resolution of 70,000 with a mass range of m/z 375-1500 and an AGC target of 3.106. MS/MS were performed in a Top 20 DDA mode. Peptides were selected for fragmentation by Higher-energy C-trap Dissociation (HCD) with a Normalized Collisional Energy of 27%, and a dynamic exclusion of 30 s. Fragment masses were measured in the Orbitrap cell at a resolution of 17,500, with an AGC target of 2.105. Monocharged peptides and unassigned charge states were excluded from the MS/MS acquisition. The maximum ion accumulation times were set to 50 msec for MS and 45 msec for MS/MS acquisitions respectively.

#### Data processing

The raw data were processed on Proteome Discoverer 2.2 or 2.4 with the mascot node (Mascot version 2.5.1) with the non-redundant protein database for *Xenopus laevis* taxonomy with a maximum of 2 missed cleavage sites. Precursor and fragment mass tolerance were set to 6 ppm and 0.02 Da respectively for Q-exactive Plus, 7 ppm and 0.5 Da respectively for Fusion. The following post-translational modifications were searched: Acetyl (Protein N-term), Oxidation (M), Phosphorylation (STY). The spectra were filtered using a 1% FDR with percolator node. For peptide identification, the data were searched against a *Xenopus laevis* database (July 2016, 17 742 entries) extracted with the NCBI protein search engine. These sequences come from various sources (UniprotKB/Swissprot, EMBL, RefSeq, GenBank, PDB, DDBJ and PIR).

### Reporting summary

Further information on research design is available in the Nature Research Reporting Summary linked to this article.

## Supplementary information

Supplementary Information

Peer Review File

Reporting Summary

## Data Availability

All data supporting the findings of this study are available within the paper and its supplementary information file. The mass spectrometry proteomics data that support the findings of this study have been deposited on the ProteomeXchange Consortium via the PRIDE partner repository with the dataset identifier PXD022739. Other relevant data are available from the corresponding author upon reasonable request. Source data are provided with this paper.

## Source data

Source Data

## References

[1] Deguchi R, Takeda N, Stricker SA (2011). Comparative biology of cAMP-induced germinal vesicle breakdown in marine invertebrate oocytes. Mol. Reprod. Dev..

[2] Jaffe, L. A. & Norris, R. P. Initiation of the meiotic prophase-to-metaphase transition in mammalian oocytes, in *Oogenesis. The universal process*. (eds. M. H. Verlhac & A. Villeneuve) 181–197 (John Wiley & Sons Ltd, Chichester; 2010).

[3] Dupre A, Daldello EM, Nairn AC, Jessus C, Haccard O (2014). Phosphorylation of ARPP19 by protein kinase A prevents meiosis resumption in Xenopus oocytes. Nat. Commun..

[4] Hara M (2012). Greatwall kinase and cyclin B-Cdk1 are both critical constituents of M-phase-promoting factor. Nat. Commun..

[5] Dupre, A. & Jessus, C. ARPP19 Phosphorylations by PKA and Greatwall: The Yin and the Yang of the Cell Decision to Divide, in *Protein Phosphorylation*. (ed. C. Prigent) 3-29 (IntechOpen, Rijeka, Croatia; 2017).

[6] Haccard O, Jessus C (2006). Redundant pathways for Cdc2 activation in Xenopus oocyte: either cyclin B or Mos synthesis. EMBO Rep..

[7] Gaffre M (2011). A critical balance between Cyclin B synthesis and Myt1 activity controls meiosis entry in Xenopus oocytes. Development.

[8] Mochida S, Ikeo S, Gannon J, Hunt T (2009). Regulated activity of PP2A-B55 delta is crucial for controlling entry into and exit from mitosis in Xenopus egg extracts. EMBO J..

[9] Lucena R, Alcaide-Gavilan M, Anastasia SD, Kellogg DR (2017). Wee1 and Cdc25 are controlled by conserved PP2A-dependent mechanisms in fission yeast. Cell Cycle.

[10] Pal G, Paraz MT, Kellogg DR (2008). Regulation of Mih1/Cdc25 by protein phosphatase 2A and casein kinase 1. J. Cell Biol..

[11] Rata S (2018). Two interlinked bistable switches govern mitotic control in mammalian cells. Curr. Biol..

[12] Lorca T (2010). Constant regulation of both the MPF amplification loop and the Greatwall-PP2A pathway is required for metaphase II arrest and correct entry into the first embryonic cell cycle. J. Cell Sci..

[13] Mochida S, Maslen SL, Skehel M, Hunt T (2010). Greatwall phosphorylates an inhibitor of protein phosphatase 2 A that is essential for mitosis. Science.

[14] Gharbi-Ayachi A (2010). The substrate of greatwall kinase, arpp19, controls mitosis by inhibiting protein phosphatase 2A. Science.

[15] Virshup DM, Shenolikar S (2009). From promiscuity to precision: protein phosphatases get a makeover. Mol. Cell.

[16] Shi Y (2009). Serine/threonine phosphatases: mechanism through structure. Cell.

[17] Castro A, Lorca T (2018). Greatwall kinase at a glance. J. Cell Sci..

[18] Kim MY (2012). Bypassing the Greatwall-Endosulfine pathway: plasticity of a pivotal cell-cycle regulatory module in Drosophila melanogaster and Caenorhabditis elegans. Genetics.

[19] Labandera AM, Vahab AR, Chaudhuri S, Kerk D, Moorhead GB (2015). The mitotic PP2A regulator ENSA/ARPP-19 is remarkably conserved across plants and most eukaryotes. Biochem. Biophys. Res. Commun..

[20] Mochida S, Hunt T (2012). Protein phosphatases and their regulation in the control of mitosis. EMBO Rep..

[21] Williams BC (2014). Greatwall-phosphorylated Endosulfine is both an inhibitor and a substrate of PP2A-B55 heterotrimers. eLife.

[22] Hached K (2019). ENSA and ARPP19 differentially control cell cycle progression and development. J. Cell Biol..

[23] Zhao Y (2008). Roles of greatwall kinase in the regulation of cdc25 phosphatase. Mol. Biol. Cell.

[24] Dupre A (2013). The phosphorylation of ARPP19 by Greatwall renders the auto-amplification of MPF independently of PKA in Xenopus oocytes. J. Cell Sci..

[25] Yu J, Zhao Y, Li Z, Galas S, Goldberg ML (2006). Greatwall kinase participates in the Cdc2 autoregulatory loop in Xenopus egg extracts. Mol. Cell.

[26] Dupre AI, Haccard O, Jessus C (2017). The greatwall kinase is dominant over PKA in controlling the antagonistic function of ARPP19 in Xenopus oocytes. Cell Cycle.

[27] Hegarat N (2020). Cyclin A triggers Mitosis either via the Greatwall kinase pathway or Cyclin B. EMBO J.

[28] Mochida S, Rata S, Hino H, Nagai T, Novak B (2016). Two bistable switches govern M phase entry. Curr. Biol..

[29] Girault JA (2012). Integrating neurotransmission in striatal medium spiny neurons. Adv. Exp. Med. Biol..

[30] Newmeyer DD, Lucocq JM, Burglin TR, De Robertis EM (1986). Assembly in vitro of nuclei active in nuclear protein transport: ATP is required for nucleoplasmin accumulation. EMBO J..

[31] Walsh DA, Ashby CD, Gonzalez C, Calkins D, Fischer EH (1971). Krebs EG: Purification and characterization of a protein inhibitor of adenosine 3’,5’-monophosphate-dependent protein kinases. J. Biol. Chem..

[32] Cohen P, Holmes CF, Tsukitani Y (1990). Okadaic acid: a new probe for the study of cellular regulation. Trends Biochem. Sci..

[33] Bialojan C, Takai A (1988). Inhibitory effect of a marine-sponge toxin, okadaic acid, on protein phosphatases. Specif. Kinet. Biochem. J..

[34] Honkanen RE (1990). Characterization of microcystin-LR, a potent inhibitor of type 1 and type 2 A protein phosphatases. J. Biol. Chem..

[35] Goris J, Hermann J, Hendrix P, Ozon R, Merlevede W (1989). Okadaic acid, a specific protein phosphatase inhibitor, induces maturation and MPF formation in Xenopus laevis oocytes. FEBS Lett..

[36] Jessus C (1991). Tyrosine phosphorylation of p34cdc2 and p42 during meiotic maturation of Xenopus oocyte. Antagonistic action of okadaic acid and 6-DMAP. Development.

[37] Frank-Vaillant M, Jessus C, Ozon R, Maller JL, Haccard O (1999). Two distinct mechanisms control the accumulation of cyclin B1 and mos in xenopus oocytes in response to progesterone. Mol. Biol. Cell.

[38] Ozon R, Belle R, Huchon D, Mulner O (1979). Roles of cyclic AMP and calcium in maturation of xenopus laevis oocytes. J. Steroid Biochem..

[39] Wang J, Liu XJ (2004). Progesterone inhibits protein kinase A (PKA) in Xenopus oocytes: demonstration of endogenous PKA activities using an expressed substrate. J. Cell Sci..

[40] Maller JL, Butcher FR, Krebs EG (1979). Early effect of progesterone on levels of cyclic adenosine 3’:5’-monophosphate in Xenopus oocytes. J. Biol. Chem..

[41] Huchon D, Ozon R, Demaille JG (1981). Protein phosphatase-1 is involved in Xenopus oocyte maturation. Nature.

[42] Rebelo S, Santos M, Martins F, da Cruz e Silva EF, da Cruz e Silva OA (2015). Protein phosphatase 1 is a key player in nuclear events. Cell. Signal..

[43] Heim A, Konietzny A, Mayer TU (2015). Protein phosphatase 1 is essential for greatwall inactivation at mitotic exit. EMBO Rep..

[44] Ma S (2016). Greatwall dephosphorylation and inactivation upon mitotic exit is triggered by PP1. J. Cell Sci..

[45] Wassmann K (2013). Sister chromatid segregation in meiosis II: deprotection through phosphorylation. Cell Cycle.

[46] Saurin AT (2018). Kinase and phosphatase cross-talk at the kinetochore. Front. cell developmental Biol..

[47] Isoda M (2011). Dynamic regulation of Emi2 by Emi2-bound Cdk1/Plk1/CK1 and PP2A-B56 in meiotic arrest of Xenopus eggs. Dev. Cell.

[48] Heim A, Tischer T, Mayer TU (2018). Calcineurin promotes APC/C activation at meiotic exit by acting on both XErp1 and Cdc20. EMBO Rep.

[49] Wang X, Bajaj R, Bollen M, Peti W, Page R (2016). Expanding the PP2A interactome by defining a B56-specific SLiM. Structure.

[50] Hertz EP (2016). A conserved motif provides binding specificity to the PP2A-B56 phosphatase. Mol. Cell.

[51] Cundell MJ (2016). A PP2A-B55 recognition signal controls substrate dephosphorylation kinetics during mitotic exit. J. Cell Biol..

[52] Moura M, Conde C (2019). Phosphatases in mitosis: roles and regulation. Biomolecules.

[53] Castilho PV, Williams BC, Mochida S, Zhao Y, Goldberg ML (2009). The M phase kinase Greatwall (Gwl) promotes inactivation of PP2A/B55delta, a phosphatase directed against CDK phosphosites. Mol. Biol. Cell.

[54] Wang F (2018). Protein interactomes of protein phosphatase 2A B55 regulatory subunits reveal B55-mediated regulation of replication protein A under replication stress. Sci. Rep..

[55] Leslie SN, Nairn AC (2019). cAMP regulation of protein phosphatases PP1 and PP2A in brain. Biochimica et. biophysica acta Mol. cell Res..

[56] Ahn JH (2007). Protein kinase A activates protein phosphatase 2A by phosphorylation of the B56delta subunit. Proc. Natl Acad. Sci. USA.

[57] Duckworth BC, Weaver JS, Ruderman JV (2002). G2 arrest in Xenopus oocytes depends on phosphorylation of cdc25 by protein kinase A. Proc. Natl Acad. Sci. USA.

[58] Han SJ, Chen R, Paronetto MP, Conti M (2005). Wee1B is an oocyte-specific kinase involved in the control of meiotic arrest in the mouse. Curr. Biol..

[59] Hemmings HC, Greengard P, Tung HY, Cohen P (1984). DARPP-32, a dopamine-regulated neuronal phosphoprotein, is a potent inhibitor of protein phosphatase-1. Nature.

[60] Bibb JA (1999). Phosphorylation of DARPP-32 by Cdk5 modulates dopamine signalling in neurons. Nature.

[61] Horiuchi A, Williams KR, Kurihara T, Nairn AC, Greengard P (1990). Purification and cDNA cloning of ARPP-16, a cAMP-regulated phosphoprotein enriched in basal ganglia, and of a related phosphoprotein, ARPP-19. J. Biol. Chem..

[62] Girault JA, Horiuchi A, Gustafson EL, Rosen NL, Greengard P (1990). Differential expression of ARPP-16 and ARPP-19, two highly related cAMP-regulated phosphoproteins, one of which is specifically associated with dopamine-innervated brain regions. J. Neurosci..

[63] Andrade EC (2017). ARPP-16 is a striatal-enriched inhibitor of protein phosphatase 2A regulated by microtubule-associated serine/threonine kinase 3 (Mast 3 kinase). J. Neurosci..

[64] Musante V (2017). Reciprocal regulation of ARPP-16 by PKA and MAST3 kinases provides a cAMP-regulated switch in protein phosphatase 2A inhibition. eLife.

[65] Margolis SS (2006). A role for PP1 in the Cdc2/Cyclin B-mediated positive feedback activation of Cdc25. Mol. Biol. Cell.

[66] Jessus C, Thibier C, Ozon R (1987). Levels of microtubules during the meiotic maturation of the Xenopus oocyte. J. Cell Sci..

[67] Laemmli UK (1970). Cleavage of structural proteins during the assembly of the head of bacteriophage T4. Nature.

[68] Dupre A, Jessus C, Ozon R, Haccard O (2002). Mos is not required for the initiation of meiotic maturation in Xenopus oocytes. EMBO J..

[69] Schagger H (2006). Tricine-SDS-PAGE. Nat. Protoc..

[70] Dulubova I (2001). ARPP-16/ARPP-19: a highly conserved family of cAMP-regulated phosphoproteins. J. Neurochem..

[71] Bosch M (1995). The PR55 and PR65 subunits of protein phosphatase 2A from Xenopus laevis. molecular cloning and developmental regulation of expression. Eur. J. Biochem..

[72] De Smedt V (2002). Thr-161 phosphorylation of Monomeric Cdc2. Regulation by protein phosphatase 2 C in Xenopus oocytes. J. Biol. Chem..

[73] Studier FW (2005). Protein production by auto-induction in high density shaking cultures. Protein Expr. Purif..

[74] Thomas J (1991). Expression in Escherichia coli and characterization of the heat-stable inhibitor of the cAMP-dependent protein kinase. J. Biol. Chem..

