# Supplementary material for: The M-phase regulatory phosphatase PP2A-B55δ opposes protein kinase A on Arpp19 to initiate meiotic division
Source: Nat Commun. 2021 Mar 23;12:1837. doi: 10.1038/s41467-021-22124-0 (PMC7988065; doi:10.1038/s41467-021-22124-0)
Supplement: Supplementary file 1 — Supplementary Information [file 41467_2021_22124_MOESM1_ESM.pdf]

The M-phase regulatory phosphatase PP2A-B55δ opposes protein kinase A on Arpp19 to initiate meiotic division.

Tom Lemonnier[1,#], Enrico Maria Daldello[1,#], Robert Poulhe[1], Tran Le[1], Marika Miot[1], Laurent Lignières[2], Catherine Jessus[1, &] and Aude Dupré[1, &,*]

[1] Sorbonne Université, CNRS, Laboratoire de Biologie du Développement - Institut de Biologie Paris Seine, LBD - IBPS, F-75005 Paris, France. [2] Université de Paris, CNRS, Institut Jacques Monod, F-75013 Paris, France.

[*]Corresponding author: aude-isabelle.dupre@upmc.fr

[#] and [&]: These authors contributed equally to this work.

Description of the supplementary information

Supplementary information includes 5 Figures and 4 Tables.

Supplementary Figure 1: GST-Arpp19 is truncated in C-terminus during expression and purification from bacteria.

Supplementary Figure 2: Biochemical isolation of S109-phosphatase from prophase extracts - Separation of fraction 6 from the Mono Q column with Phenyl-Superose and Superose 12 columns.

Supplementary Figure 3: Arpp19 is dephosphorylated at both S109 and S67 in prophase extracts – PP2A depletion in prophase extracts.

Supplementary Figure 4: Cter-GST-Arpp19 is phosphorylated at S109 by PKA and does not affect meiosis resumption.

Supplementary Figure 5: Endogenous Arpp19 is partially dephosphorylated at S109 one hour after progesterone stimulation.

Supplementary Table 1: LC-MS/MS analysis of the S109-phosphatase fractions from the biochemical isolation illustrated in Figures 3, 4, 5 and S2.

Supplementary Table 2: LC-MS/MS analysis of the S109-phosphatase enriched fractions from Experiment 2.

Supplementary Table 3: LC-MS/MS analysis of the S109-phosphatase enriched fractions from Experiment 3.

Supplementary Table 4: List of primers used to clone ^{gst}Cter-GST-Arpp19, His-PP1, GST-B55δ and His-B55δ.

Supplementary Figure 1

a

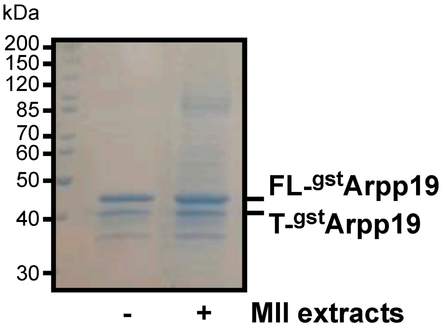

b

FL-^{gst}Arpp19 (peptide analysis of FL-^{gst}-Arpp19 sample)

msgenqetkaqeessaleqkeiddkvvspekseeiklkarypnlgpkpggsdflrkrlqkgqkyfdsgdynmakakmknkqlptaasdktevtgdhiptpqdlpqrkpslvasklag
[k].aqeessaleqk.[e]
[k].aqeessaleqkeiddk.[v]
[k].aqeessaleqkeiddkvvspek.[s]
[k].eiddkvvspek.[s]
[k].eiddkvvspekseeik.[l]
[k].vvspekseeik.[l]
[k].arypnlgpkpggsdflr.[k]
[r].ypnigpkpggsdflrk.[r]
[r].ypnigpkpggsdflr.[k]
[k].gqkyfdsgdynmak.[a]
[k].yfdsgdynmakak.[im]
[k].yfdsgdynmak.[a]
[k].qlptaasdk.[t]
[k].qlptaasdktevtgdhiptpqdlpqr.[k]
[k].nkqlptaasdktevtgdhiptpqdlpqr.[k]
[k].qlptaasdktevtgdhiptpqdlpqrkpslvask.[l]
[k].tevtgdhiptpqdlpqrkpslvask.[l]
[k].tevtgdhiptpqdlpqr.[k]
[r].kpslvask.[l]

FL-^{gst}Arpp19 (peptide analysis of T-^{gst}Arpp19)

msgenqetkaqeessaleqkeiddkvvspekseeiklkarypnlgpkpggsdflrkrlqkgqkyfdsgdynmakakmknkqlptaasdktevtgdhiptpqdlpqrkpslvasklag
[k].aqeessaleqk.[e]
[k].aqeessaleqkeiddk.[v]
[k].aqeessaleqkeiddkvvspek.[s]
[k].eiddkvvspek.[s]
[k].eiddkvvspekseeik.[l]
[k].vvspekseeik.[l]
[k].vvspekseeiklk.[a]
[k].arypnlgpkpggsdflr.[k]
[r].ypnigpkpggsdflrk.[r]
[r].ypnigpkpggsdflr.[k]
[k].yfdsgdynmakak.[im]
[k].yfdsgdynmak.[a]
[k].qlptaasdktevtgdhiptpqdlpqr.[k]
[k].tevtgdhiptpqdlpqr.[k]

Legend. GST-Arpp19 is truncated in C-terminus during expression and purification from bacteria. (a) GST-Arpp19 coupled to sepharose GSH-beads was phosphorylated or not in metaphase II (MII) extracts. Proteins bound to beads were subjected to a 10% SDS gel electrophoresis. After Coomassie staining, two major bands were detected, the upper and most abundant one corresponding to the molecular weight expected for full-length GST-Arpp19 (FL-^{gst}Arpp19) and a lower one that could correspond to a truncated form of Arpp19 (T-^{gst}Arpp19). The preparative stained gel was prepared one time prior its analysis. kDa: kiloDalton. **(b)** Each band was cut out from the polyacrylamide gel and analyzed by LC-MS/MS. The sequences of peptides identified by LC-MS/MS analysis from either FL-^{gst}Arpp19 or T-

gstArpp19 are aligned with *Xenopus* Arpp19 sequence. The C-terminal peptide, PSLVASKLAG, is never recovered from T-gstArpp19. When GST-Arpp19 has been phosphorylated in MII extracts, phosphorylated S109 (in red and underlined) is detected by LC-MS/MS in some peptides generated by FL-gstArpp19 but never in the peptides generated by T-gstArpp19. In contrast, phosphorylated S67 (in blue and underlined) is detected in peptides generated by both FL-gstArpp19 and T-gstArpp19. Source data are provided as a Source Data file.

Method. Bacterially expressed GST-Arpp19 was bound to Sepharose GSH-beads[1]. GST-Arpp19 coupled to beads was incubated or not in metaphase II extracts to generate the double S167-S109 phosphorylated form of Arpp19. Sepharose beads coupled to GST-Arpp19 were subjected to a 10% SDS gel electrophoresis[2]. After Coomassie staining, the bands were cut out from the gel. Gel plugs were discolored using 50 mM ACN/NH_4HCO_3 (50/50) for 15 min under agitation. Plugs were reduced with a solution of 10 mM DL-Dithiothreitol for 45 min at 56°C, and then alkylated using 55 mM Iodoacetamide for 45 min at room temperature. Digestion of samples, LC-MS/MS data acquisition on Q-exactive Plus instrument and processing were performed as described in the Method section. Samples were also analyzed using an Orbitrap Fusion, coupled to a Nano-LC Proxeon equipped with an easy spray ion source (Thermo Scientific). On the Orbitrap Fusion instrument, peptides were loaded with an online preconcentration method and separated by chromatography using a Pepmap-RSLC C18 column (0.75 x 750 mm, 2 ⌠m, 100 Å) from Thermo Scientific, equilibrated at 50°C and operated at a flow rate of 300 nl/min. Peptides were eluted by a gradient of solvent A (H_2O, 0.1% FA) and solvent B (ACN/H_2O 80/20, 0.1% FA), the column was first equilibrated 5 min with 95% of A, then B was raised to 28% in 105 min and to 40% in 15 min. Finally, the column was washed with 95% B during 20 min and re-equilibrated at 95% A during 10 min. Peptides masses were analyzed in the Orbitrap cell in full ion scan mode, at a resolution of 120,000, a mass range of m/z 350-1550 and an AGC target of 4.105. MS/MS were performed in the top speed 3s mode. Peptides were selected for fragmentation by Higher-energy C-trap Dissociation (HCD) with a Normalized Collisional Energy of 27% and a dynamic exclusion of 60 sec. Fragment masses were measured in an Ion trap in the rapid mode, with an AGC target of 1.104. Monocharged peptides and unassigned charge states were excluded from the MS/MS acquisition. The maximum ion accumulation times were set to 100 msec for MS and 35 msec for MS/MS acquisitions respectively.

Data availability: The data have been deposited on the ProteomeXchange Consortium via the PRIDE partner repository with the dataset identifier PXD022739 (https://www.ebi.ac.uk/pride/profile/reviewer_pxd022739).

Supplementary Figure 2

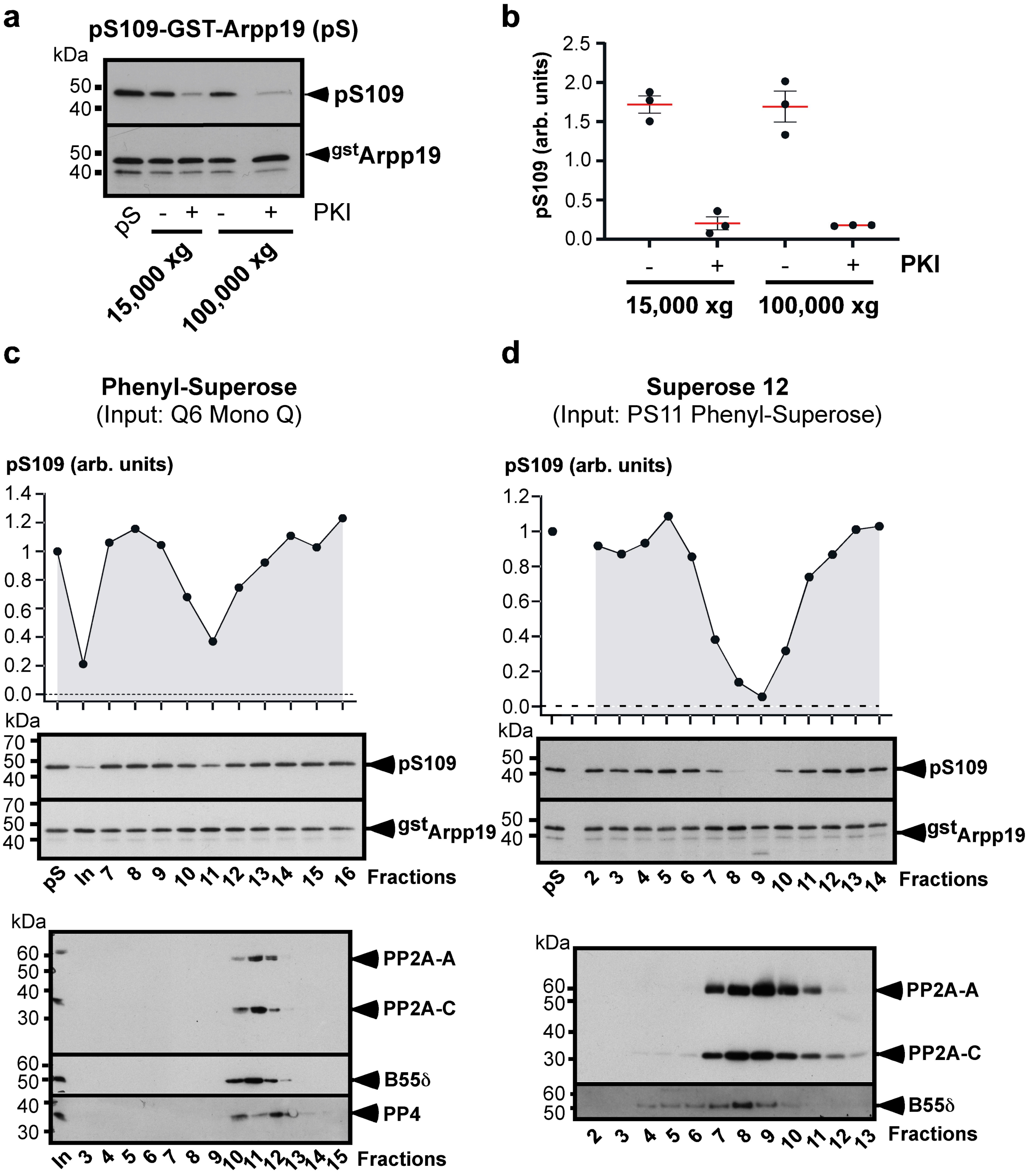

Legend. Biochemical isolation of S109-phosphatase from prophase extracts - Separation of fraction 6 from the Mono Q column with Phenyl-Superose and Superose 12 columns. (**a**) Prophase oocytes were lysed and centrifuged at 15,000 xg. The supernatant was then ultracentrifuged or not at 100,000 xg. S109-phosphatase activity was assayed in the 15,000 xg and 100,000 xg supernatants supplemented or not with PKI using pS109-GST-Arpp19 as a substrate (pS for phosphorylated substrate). S109 phosphorylation of GST-Arpp19 (pS109) and total GST-Arpp19 (^{gst}Arpp19) were western blotted using respectively

phospho-S109-Arpp19 and GST antibodies. The experiment was repeated 3 times with similar results. (**b**) Quantifications of S109 phosphorylation from 3 independent experiments performed as in (a). Data are shown as mean (red bars) +/- SEM. Each dot represents one experiment. (**c-d**) Continuation of experiment illustrated in Fig. 3. "pS": phosphorylated starting pS109-GST-Arpp19 substrate. "In": input sample loaded on the column. S109 phosphorylation quantification: an arbitrary unit of 1 was attributed to the phosphorylation level of S. (**c**) Phenyl-Superose. Fraction 6 from the Mono Q column (see Fig. 3c) was loaded on the column. Elution profile of S109-phosphatase activity after Phenyl-Superose column and western blot analysis of fractions 3 to 15 with antibodies directed against catalytic subunits of PP2A (PP2A-C) and PP4, PP2A scaffold subunit A (PP2A-A) and PP2A regulatory subunit B55δ. (**d**) Superose 12. Fraction 11 from the Phenyl-Superose column (see c) was loaded on the column. Elution profile of S109-phosphatase activity after Superose 12 column and western blot analysis of fractions 2 to 13 with antibodies directed against PP2A scaffold subunit (PP2A-A), PP2A catalytic subunit (PP2A-C) and PP2A regulatory subunit B55δ. kDa: kiloDalton. arb. units: arbitrary units. Source data are provided as Source Data file.

Supplementary Figure 3

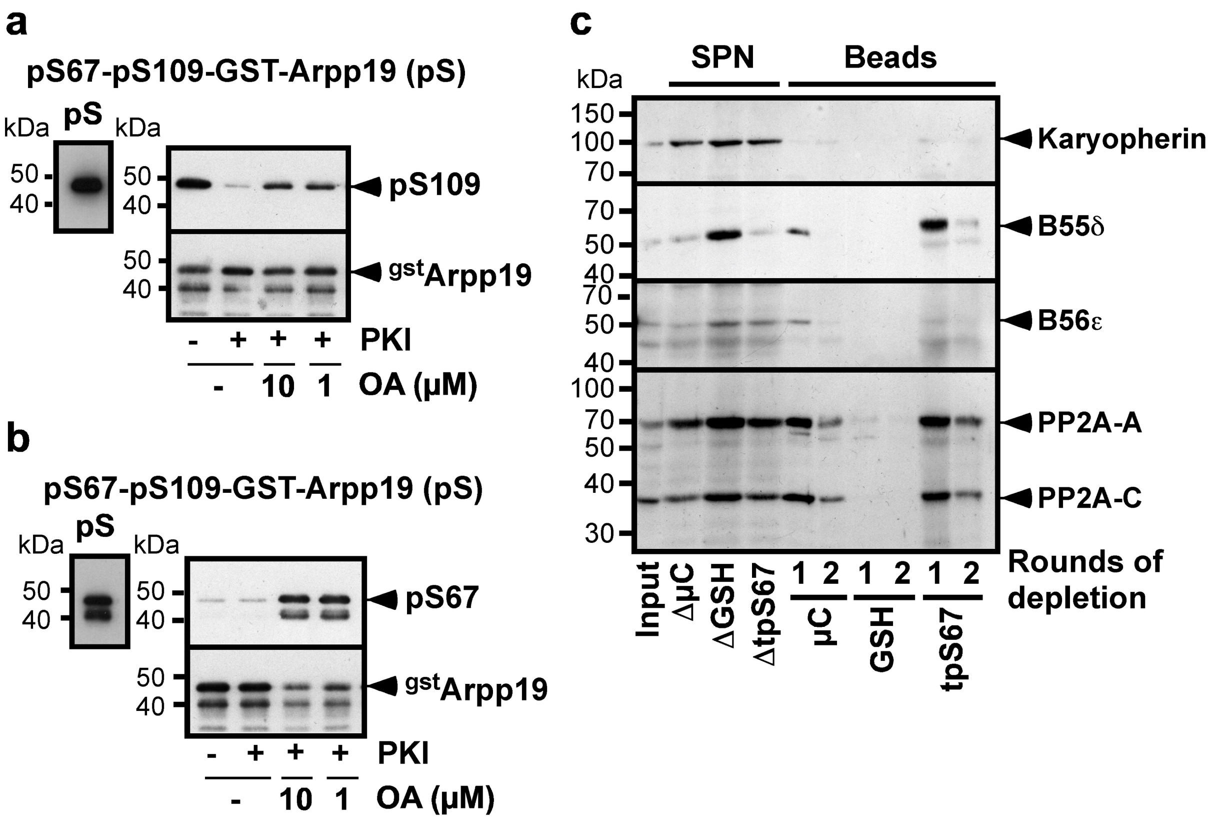

Legend. Arpp19 is dephosphorylated at both S109 and S67 in prophase extracts – PP2A depletion in prophase extracts. (**a-b**) Prophase extracts were supplemented or not with PKI and further incubated with a double S109-S67 phosphorylated GST-Arpp19 (pS67-pS109-GST-Arpp19 or "pS"), in the presence or not of either 1 µM or 10 µM okadaic acid (OA). The phosphorylation of GST-Arpp19 at either S109 (**a**) or S67 (**b**) was analyzed by western blot with antibodies directed against S109-phosphorylated Arpp19 (pS109) or S67-phosphorylated Arpp19 (pS67). Total GST-Arpp19 (^{gst}Arpp19) was western blotted with the anti-GST antibody. The experiment was repeated 3 times with similar results. (**c**) Prophase extracts were incubated twice with either microcystin beads (µC), GSH-beads (GSH) or GST-Arpp19 thiophosphorylated at S67 coupled to GSH-beads (tpS67). After separation, the supernatants (SPN) resulting from the two incubation rounds with µC (ΔµC), GSH (ΔGSH) or tpS67 (ΔtpS67) and the beads were analyzed by western blot with antibodies directed against karyopherin (SC-1863, Santa Cruz, 1:500) as control, PP2A catalytic subunit (PP2A-C) and PP2A-regulatory subunits A (PP2A-A), B55δ and B56ε. The experiment was repeated 3 times with similar results. "Input": prophase extracts before the addition of beads. kDa: kiloDalton. Source data are provided as a Source Data file.

Method. Production of pS67-pS109-GST-Arpp19. GST-Arpp19 was purified as described in[1], except that it was not eluted from Sepharose GSH-beads at the end of the procedure.

GST-Arpp19 coupled to beads was doubly phosphorylated and then used as a substrate of S109- and S67-phosphatase activities in prophase extracts as described in the Method section.

Supplementary Figure 4

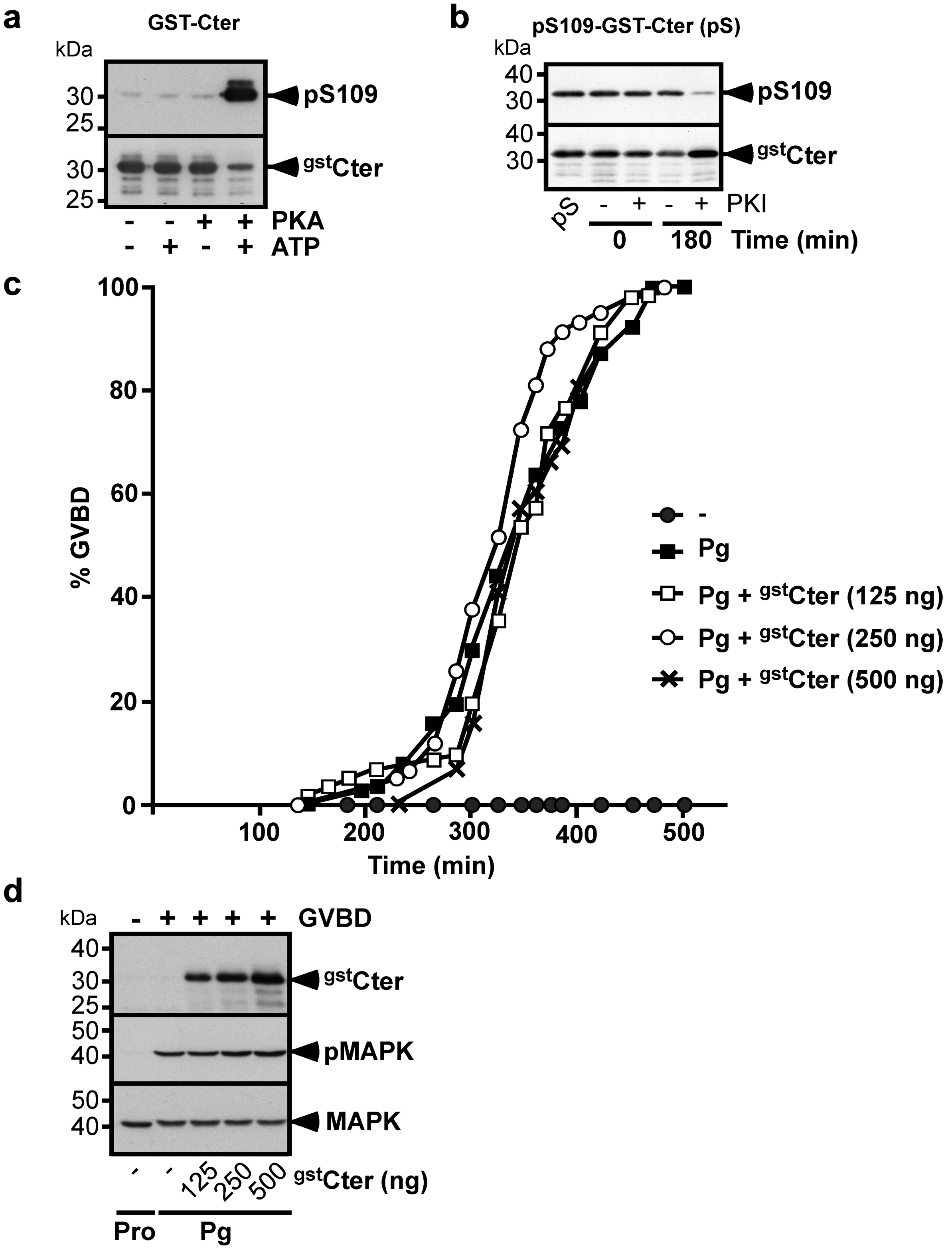

Legend. Cter-GST-Arpp19 is phosphorylated at S109 by PKA and does not affect meiosis resumption. (**a**) Cter-GST-Arpp19 was incubated with or without PKA catalytic subunit in the presence or in the absence of ATP. S109 phosphorylation of Cter-GST-Arpp19 (pS109) and total Cter-GST-Arpp19 (^{gst}Cter) were western blotted using phospho-S109-

Arpp19 and GST antibodies. The experiment was repeated 3 times with similar results. (**b**) Cter-GST-Arpp19 was *in vitro* phosphorylated by PKA and then incubated in prophase extracts, supplemented or not with PKI for 3h. S109 phosphorylation of Cter-GST-Arpp19 (pS109) and total Cter-GST-Arpp19 (^{gst}Cter) were western blotted using respectively phospho-S109-Arpp19 and GST antibodies. pS: phosphorylated pS109-GST-Cter substrate. The experiment was repeated 3 times with similar results. (**c**) Prophase oocytes were injected with various amounts of Cter-GST-Arpp19 (^{gst}Cter) as indicated, and stimulated with progesterone (Pg). GVBD was scored as a function of time. (**d**) Same experiment as in (c). Prophase (Pro) or progesterone-stimulated oocytes (Pg), injected or not with Cter-GST-Arpp19 (^{gst}Cter), were collected at the time of GVBD. Cter-GST-Arpp19 was pulled-down and western blotted with the anti-GST antibody. Total oocyte extracts were western blotted with antibodies against phosphorylated MAPK (pMAPK) and total MAPK. The experiment was repeated 3 times with similar results. kDa: kiloDalton. Source data are provided as a Source Data file.

Supplementary Figure 5.

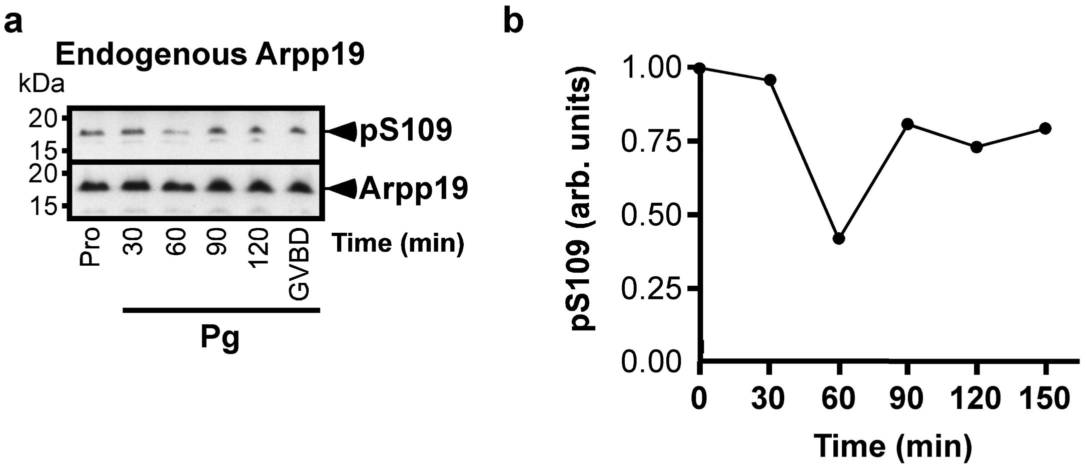

Legend. Endogenous Arpp19 is partially dephosphorylated at S109 one hour after progesterone stimulation. (**a**) Prophase oocytes (Pro) were stimulated or not with progesterone (Pg) and collected at the indicated time. GVBD occurred at 150 min in this experiment. Oocytes were collected at the indicated times after Pg addition and western blotted with antibodies against S109-phosphorylated Arpp19 (pS109) and total Arpp19. (**b**) S109 phosphorylation of the experiment illustrated in (a) was quantified. Time “0”: prophase oocyte. The experiment was repeated 7 times with similar results. kDa: kiloDalton. arb. units: arbitrary units. Source data are provided as a Source Data file.

Supplementary Table 1

					Mascott scores											
						From Q5				From Q6				From Q7-8		
Family	Description	Accession	Gene Symbol	Coverage (%)	Q5	PS 11	S8	S9	Q6	PS 11	S8	S9	Q7-8	PS 10-11	S10	S11
PP2A-A	Structural subunit Aβ	148230849	ppp2r1b; ppp2r1b.S	78	2086	5660	4634	5504	2764	9434	7779	4015	3273	3644	5333	2579
	Structural subunit Aα	148222150	ppp2r1a-b; ppp2r1a.S	82	2201	4326	3524	4524	2122	7476	4180	2097	2325	2632	3411	1351
	Structural subunit Aα	148224496	ppp2r1a-a; ppp2r1a.L	81	1917	4171	3297	4108	2099	7265	4094	2030	2220	2549	3347	1302
PP2A-C	Catalytic subunit Cα	148230509	ppp2ca; ppp2ca.L	89	1521	3288	2771	2870	1788	6924	3284	1889	1527	952	1296	831
	Catalytic subunit Cβ	148234623	ppp2cb; ppp2cb.L	89	1476	3191	2877	2817	1790	7260	3336	1932	1562	951	1405	842
B55	Regulatory subunit Bα	148234757	ppp2r2a; ppp2r2a.S	79	818	3241	240	414	969	3338	476	166	377	834	39	51
	Regulatory subunit Bδ	147900119	ppp2r2d; ppp2r2d.S	62	1028	3330	109	287	1023	4991	277	176	165	788	80	67
	Regulatory subunit Bβ	148231951	ppp2r2b; ppp2r2b.L	23	275	1052	87	120	319	1418	151	43	64	308	29	48
B56	Regulatory subunit B'α	168693595	ppp2r5a; ppp2r5a.S	54					19	50			779	1057	639	272
	Regulatory subunit B'β	148234627	ppp2r5b; ppp2r5b.L	50					19	40			721	803	489	195
	Regulatory subunit B'γ	148236119	ppp2r5c; ppp2r5c.L	40	71	324			131	141			125	105	121	113
	Regulatory subunit B'ε	148236023	ppp2r5e; ppp2r5e.S	51	34				27				700	491	827	485
	Regulatory subunit B'ε	147902694	ppp2r5e.L	54	34				27				546	462	901	623
PP1	Catalytic subunit Cα	147903539	ppp1ca; ppp1ca.L	69	111				906	41			1589	44	17	22
	Catalytic subunit Bγ	148224548	ppp1cc-B, ppp1cc.S	64	203				752				1308	60	15	
	Catalytic subunit Cβ	148224494	ppp1cb.L	64	161				775	30			1164	41	15	
PP1-R	Regulatory subunit 11	148229062	ppp1r11; ppp1r11.L	38					33				224			
PP2C	PP1A	147905165	ppm1a; ppm1a.L	90	14	223		28	57	290			1484	3921	8960	8659
	Mg2+/Mn2+ dependent, 1B	148227634	ppm1b; ppm1b.L	31	14	99				144			310	1354	3181	3049
PP3	Catalytic subunit Cα	148235473	ppp3ca; ppp3ca.L	31	57	176			62	85			176	88		
PP4	Catalytic subunit	148226861	ppp4c.S	17	173		25	143	320	539	58	60	517	34	75	0
PP6	Catalytic subunit	147906292	ppp6c; ppp6c.L	6					33							

Legend. LC-MS/MS analysis of the S109-phosphatase fractions from the biochemical isolation. Same experiment as in Figs. 3 to 5 and Supplementary Fig. 3. Proteins present in the fractions with active S109-phosphatase after the Mono Q column (Q5, Q6 and Q7-8 -a pool of 7 and 8-), after the Phenyl-Superose column (PS11 from Q5, PS11 from Q6 and PS10-11 from Q7-8) and after Superose 12 column (S8 and S9 from Q5>PS11, S8 and S9 from Q6>PS11, S10 and S11 from Q7-8>PS10-11) were identified by LC-MS/MS analysis. The table lists proteins carrying a phosphatase enzymatic activity and their regulators. PP1-R: PP1 regulator. Data availability: The data have been deposited on the ProteomeXchange Consortium via the PRIDE partner repository with the dataset identifier PXD022739 (https://www.ebi.ac.uk/pride/profile/reviewer_pxd022739).

Supplementary Table 2

					Mascot scores			
Family	Description	Accession	Gene Symbol	Coverage (%)	S5	S7	S8	S9
PP2A-A	Structural subunit Aβ	148230849	ppp2r1b; ppp2r1b.S	60	810	2675	2392	1509
	65 kDa regulatory subunit A β isoform-like	148227844	LOC398563	58	829	2625	2458	1509
	Structural subunit Aα	148222150	ppp2r1a-b; ppp2r1a.S	52	650	1380	1566	706
	Structural subunit Aα	148224496	ppp2r1a-a; ppp2r1a.L	52	562	1205	1438	764
PP2A-C	Catalytic subunit Cα	148230509	ppp2ca; ppp2ca.L	56	399	1332	1014	472
	Catalytic subunit Cβ	148234623	ppp2cb; ppp2cb.L	53	399	1306	1003	472
B55	Regulatory subunit Bδ	147900119	ppp2r2d; ppp2r2d.S	34	65	171	296	233
	Regulatory subunit Bα	148234757	ppp2r2a; ppp2r2a.S	33	66	324	403	427
B56	Regulatory subunit B'α	168693595	ppp2r5a; ppp2r5a.S	51	317	761	707	404
	Regulatory subunit B'ε	148236023	ppp2r5e; ppp2r5e.S	32	35	226	181	126
		147902694	ppp2r5e.L	26	35	251	138	105
	Regulatory subunit B'γ	148236119	ppp2r5c; ppp2r5c.L	8		105	70	43
PP2C	PP1A	147905165	ppm1a; ppm1a.L	26				184
	Mg2+/Mn2+ dependent, 1B	148227634	ppm1b; ppm1b.L	18				164
PP1	Catalytic subunit Cα	147903539	ppp1ca; ppp1ca.L	9	67	35	34	36
PP3	Catalytic subunit Cα	148235473 or 148235616*	ppp3ca; ppp3ca.L ppp3ca.S	9	82		62	108
PP4	Catalytic subunit	148226861	ppp4c.S	13		113	60	37
PP6	Catalytic subunit	147906292	ppp6c; ppp6c.L	3	23			
S109-phosphatase activity					-	-	+	-

*: The sequence of the identified peptides does not allow to distinguish between these two isoforms.

Legend. LC-MS/MS analysis of the S109-phosphatase enriched fractions from experiment 2 – Analysis of Superose fractions (Input: pool of Q5 to 8 > PS11). The isolation procedure of S109-phosphatase was repeated 4 times with oocytes collected from different females. The results of the experiments 1, 2 and 3 are shown in Supplementary Tables 1, 2 and 3 respectively. Experiment 4 was analyzed by western blot but not by LC-MS/MS. In experiment 2, S109-phosphatase activity was recovered in fractions Q5 to Q8 after the Mono Q column. These fractions were pooled before loading on Phenyl-Superose column. In experiment 3, S109-phosphatase activity was recovered in a single fraction after the Mono Q column, Q8, which was further loaded on the Phenyl-Superose column. S109-phosphatase activity was recovered in PS11 after the Phenyl-Superose column. PS11 was loaded on the Superose 12 column. The phosphatase content was estimated by LC-MS/MS sequencing in fractions from Superose 12 column. S109-phosphatase activity is indicated with "+" or "-". Data availability: The data have been deposited on the ProteomeXchange Consortium via the PRIDE partner repository with the dataset identifier PXD022739 (https://www.ebi.ac.uk/pride/profile/reviewer_pxd022739).

Supplementary Table 3

					Mascot scores						
					PS	Superose fractions					
Family	Description	Accession	Gene symbol	Coverage (%)	PS11	S5	S6	S7	S8	S9	S10
PP2A-A	65 kDa regulatory subunit Aβ isoform-like	148227844	LOC398563	52,63157895	1888	388	251	415	620	838	403
	Structural subunit Aβ	148230849	ppp2r1b; ppp2r1b.S	52,12224109	1825	347	255	395	572	814	403
	Structural subunit Aα	148222150	ppp2r1a-b; ppp2r1a.S	42,44482173	1181	141	51	290	370	454	218
	Structural subunit Aα	148224496	ppp2r1a-a; ppp2r1a.L	41,93548387	1135	141	51	321	399	484	218
PP2A-C	Catalytic subunit Cα	148230509	ppp2ca; ppp2ca.L	53,07443366	721	171	69	108	214	227	99
	Catalytic subunit Cβ	148234623	ppp2cb; ppp2cb.L	50,48543689	690	155	69	132	233	243	99
B55	Regulatory subunit Bα	148234757	ppp2r2a; ppp2r2a.S	46,17117117	866	56	67	106	81	151	42
	Regulatory subunit Bδ	147900119	ppp2r2d; ppp2r2d.S	27,74049217	706	65	88	88	66	71	33
B56	Regulatory subunit B'α	168693595	ppp2r5a; ppp2r5a.S	23,52941176	438					43	
	Regulatory subunit B'β	148234627	ppp2r5b; ppp2r5b.L	26,2605042	452	41				43	
	Regulatory subunit B'ε	148236023	ppp2r5e; ppp2r5e.S	18,84368308	268						
		147902694	ppp2r5e.L	17,55888651	249						
	Regulatory subunit B'γ	148236119	ppp2r5c; ppp2r5c.L	4,347826087	30						
PP2C	PP1A	147905165	ppm1a; ppm1a.L	46,73629243	1011		41	31		218	389
	Mg2+/Mn2+ dependent, 1B	148227634	ppm1b; ppm1b.L	15,42168675	549		24			81	139
PP1	Catalytic subunit Bγ	148224548	ppp1cc.S, ppp1cc-B	23,043	206						
PP3	Catalytic subunit Cα	148235473 or 148235616*	ppp3ca; ppp3ca.L ppp3ca.S	5,598455598	94						
PP6	Catalytic subunit	147906292	ppp6c; ppp6c.L	10,16393443	15						
PP4-R	Regulatory subunit 2-B	147901735	ppp4r2; ppp4r2.S	3,768844221	19						
S109-phosphatase activity					+++	-	+	++	++	+	-

*: The sequence of the identified peptides does not allow to distinguish between these two isoforms.

Legend. LC-MS/MS analysis of the S109-phosphatase enriched fractions from experiment 3 - Analysis of PS11 and Superose fractions (input: Q8 > PS11). S109-phosphatase activity was recovered in a single fraction after the Mono Q column, Q8, which was further loaded on the Phenyl-Superose column. S109-phosphatase activity was recovered in PS11 after the Phenyl-Superose column. PS11 was loaded on the Superose 12 column. The phosphatase content was estimated by LC-MS/MS sequencing in PS11 and in fractions from Superose 12 column. S109-phosphatase activity is indicated with "+" or "-". Data availability: The data have been deposited on the ProteomeXchange Consortium via the PRIDE partner repository with the dataset identifier PXD022739 (https://www.ebi.ac.uk/pride/profile/reviewer_pxd022739).

Supplementary Table 4

Cter-GST-Arpp19 (pGex-6P1)	
Cter-forward	CTAGCGTAGAATTCGATATGGGGGACTACAATATGGCTAAAG
Cter-reverse	GTATCGATAAGCTTGATTCAGCCAGCCAGTTTGC
His-PP1(pRN3)	
His-PP1-forward	GATCACAGGAATTCTCCACCATGCATCATCATCATCATCATATGGGGGACGGAGAAAAACTAAATATCGACTCC
His-PP1-reverse	TACTAGCGTAGCGGCCGCTCATTTGGACTGTTTGTTTTTGTTCCGTGG
GST-B55δ (pGex-6P1)	
GST-B55δ-forward	GTTGGAATTCCCATGGCAGGAGTGG
GST-B55δ-reverse	CCCCTCGAGGGATACTCTAGAG
His-B55δ (pRN3)	
His-B55δ-forward<br>Kozak-His-B55δ-forward	TGGCAGATCTTAATGCATCATCATCATCATCATATGGCAGGACTGGGCGGAGGGAACGAT<br>TGGCAGATCTTACCACCATGCATCATCATCATCATCAT
His-B55δ-reverse	CCCCTCGAGGGATACTCTAGAG

Legend. Sequences of the primers used to clone Cter-GST-Arpp19, His-PP1 and His-B55δ. The vectors are indicated in brackets after the protein name. The cDNAs encoding either *Xenopus* catalytic PP1α or B55δ subunit were purchased from Thermo Fisher (ppp1ca 379914 Clone ID: 4682749 MXL1736-202771954) and GE healthcare (XCG ppp2cb cDNA cl5073873). PP1 was cloned into a pRN3 vector by PCR using primers His-PP1-forward encoding N-terminus Histidine-tag and His-PP1-reverse. The cDNA encoding the Cter-Arpp19 (amino-acids 68-117 of Arpp19) was cloned into a pGEX-6P-1 vector by PCR using primers Cter-forward and Cter-reverse. B55δ was first cloned into pGex-6P1 by PCR using primers GST-B55δ-forward and GST-B55δ-reverse and then subcloned into pRN3 vector using primers His-B55δ-forward encoding N-terminus Histidine-tag and His-B55δ-reverse. A kozak sequence was further inserted by PCR using primers Kozak-His-B55δ-forward and His-B55δ-reverse. All cDNAs were sequenced before further use.

References

1. Dupre, A., Daldello, E.M., Nairn, A.C., Jessus, C. & Haccard, O. Phosphorylation of ARPP19 by protein kinase A prevents meiosis resumption in Xenopus oocytes. *Nature communications* **5**, 3318 (2014).

2. Laemmli, U.K. Cleavage of structural proteins during the assembly of the head of bacteriophage T4. *Nature* **227**, 680-685 (1970).
