# Supplementary material for: The M-phase regulatory phosphatase PP2A-B55δ opposes protein kinase A on Arpp19 to initiate meiotic division
Source: Nat Commun. 2021 Mar 23;12:1837. doi: 10.1038/s41467-021-22124-0 (PMC7988065; doi:10.1038/s41467-021-22124-0)
Supplement: Supplementary file 3 — Reporting Summary [file 41467_2021_22124_MOESM3_ESM.pdf]

nature research

Corresponding author(s): DBPR - NCOMMS-19-35179B

Last updated by author(s): Feb 3, 2021

Reporting Summary

Nature Research wishes to improve the reproducibility of the work that we publish. This form provides structure for consistency and transparency in reporting. For further information on Nature Research policies, seeAuthors & Referees and theEditorial Policy Checklist .

Statistics

For all statistical analyses, confirm that the following items are present in the figure legend, table legend, main text, or Methods section.

n/a	Confirmed	
☐	☒	The exact sample size (*n*) for each experimental group/condition, given as a discrete number and unit of measurement
☐	☒	A statement on whether measurements were taken from distinct samples or whether the same sample was measured repeatedly
☐	☒	The statistical test(s) used AND whether they are one- or two-sided<br>*Only common tests should be described solely by name; describe more complex techniques in the Methods section.*
☒	☐	A description of all covariates tested
☒	☐	A description of any assumptions or corrections, such as tests of normality and adjustment for multiple comparisons
☐	☒	A full description of the statistical parameters including central tendency (e.g. means) or other basic estimates (e.g. regression coefficient) AND variation (e.g. standard deviation) or associated estimates of uncertainty (e.g. confidence intervals)
☐	☒	For null hypothesis testing, the test statistic (e.g. *F*, *t*, *r*) with confidence intervals, effect sizes, degrees of freedom and *P* value noted<br>*Give P values as exact values whenever suitable.*
☒	☐	For Bayesian analysis, information on the choice of priors and Markov chain Monte Carlo settings
☒	☐	For hierarchical and complex designs, identification of the appropriate level for tests and full reporting of outcomes
☒	☐	Estimates of effect sizes (e.g. Cohen's *d*, Pearson's *r*), indicating how they were calculated

Our web collection on statistics for biologists contains articles on many of the points above.

Software and code

Policy information about availability of computer code

Data collection	Adobe photoshop CS5 extended (version 12.0 x64)
Data analysis	Adobe photoshop CS5 extended (version 12.0 x64)<br>Image J software (version 1.51)<br>Excel Pack Office (version 16.16.3)<br>Mascot search engine (version 2.5.1)<br>Proteome Discoverer 2.2 or 2.1 (Thermo Fisher Scientific)<br>Prism 8

For manuscripts utilizing custom algorithms or software that are central to the research but not yet described in published literature, software must be made available to editors/reviewers. We strongly encourage code deposition in a community repository (e.g. GitHub). See the Nature Research guidelines for submitting code & software for further information.

Data

Policy information about availability of data

All manuscripts must include a data availability statement. This statement should provide the following information, where applicable:

- Accession codes, unique identifiers, or web links for publicly available datasets
- A list of figures that have associated raw data
- A description of any restrictions on data availability

The mass spectrometry proteomic data that support the findings of this sudy have been deposited on the ProteomeXchange Consortium via the PRIDE partner repository with the dataset identifier PXD022739. Data are available on PRIDE using the username: reviewer_pxd022739@ebi.ac.uk and the password: ey11xzGs. Peptides were identified using the Refseq non-redundant database of Xenopus laevis from NCBI (July 2016). All the other relevant data are available from the authors.

Field-specific reporting

Please select the one below that is the best fit for your research. If you are not sure, read the appropriate sections before making your selection.

☒ Life sciences ☐ Behavioural & social sciences ☐ Ecological, evolutionary & environmental sciences

For a reference copy of the document with all sections, see nature.com/documents/nr-reporting-summary-flat.pdf

Life sciences study design

All studies must disclose on these points even when the disclosure is negative.

Sample size	The number of oocytes used in experiments varies between 15 to 80 oocytes. For each experiment, the same number of oocytes was used per condition. The same volume of extracts was used in every experiments.
Data exclusions	Data were not excluded from the analysis.
Replication	The isolation procedure has been performed 4 times and the mass spectrometry analysis has been done 3 times (Supplementary Tables 1 and 2). Each experiment using either oocytes extracts or intact oocytes has been repeated at least 3 times. Representative experiments are illustrated in the manuscript, as well as quantifications of data from at least 3 replicate experiments.
Randomization	The experiments were randomized. Extracts or intact oocytes used in each experiment were collected from one single female. Control and treated oocytes/extracts come from the same female. Each experiment was repeated using oocytes from different females.
Blinding	The results of each experiment were collected and analyzed by the person in charge of the experiment. All the quantifications of western blots have been done independently by three authors, one of them ignoring the nature and the order of the loaded samples. Results were then analyzed and discussed with all the authors of the manuscript.

Reporting for specific materials, systems and methods

We require information from authors about some types of materials, experimental systems and methods used in many studies. Here, indicate whether each material, system or method listed is relevant to your study. If you are not sure if a list item applies to your research, read the appropriate section before selecting a response.

Materials & experimental systems

n/a	Involved in the study	
☐	☒	Antibodies
☒	☐	Eukaryotic cell lines
☒	☐	Palaeontology
☐	☒	Animals and other organisms
☒	☐	Human research participants
☒	☐	Clinical data

Methods

n/a	Involved in the study	
☒	☐	ChIP-seq
☒	☐	Flow cytometry
☒	☐	MRI-based neuroimaging

Antibodies

Antibodies used	Total Arpp19 (1:1,000, gift of Dr Angus Nairn, Yale University, USA), S109-phosphorylated Arpp19 (1:500), S67-phosphorylated Arpp19 (1:1,000), MAP kinase (1:1,000, Santa Cruz SC-154, clone C14, Lot #CO711), phosphorylated MAP kinase (1:1,000, Cell Signaling 9106, clone E10, Lot #45), PKA catalytic subunit (1:1,000, Abcam ab26322, Lot #GR79447-5), PKI (1:1,000, Abcam ab122816, Lot #GR46104-4), PP1 catalytic subunit (1:100, gift of Dr M. Bollen, KU Leuven, Belgium), PP2A-C catalytic subunit (1:500), PP2A-A scaffold subunit (1:500), B55δ regulatory subunit (1:500, gift of Dr S. Mochida, Kumamoto University, Japan), B56ε regulatory subunit (1:500, gift of Dr S. Mochida, Kumamoto University, Japan), PP4 catalytic subunit (1:500, Abcam ab115741, Lot #GR92913-4), PP5 catalytic subunit (1:500, gift of Dr S. Mochida, Kumamoto University, Japan), PP6 catalytic subunit (1:500, Bethyl A300-844A), Karyopherin (1:500, Sc-1863) and GST (1:10,000, Sigma A-7340).
Validation	All primary antibodies have been used to western blot either lysates from Xenopus oocytes or fractions from the isolation procedure. The validation of the antibodies can be found either on the manufacturer's website or in the listed articles.<br><br>Total Arpp19:<br>• Dulubova, I., Horiuchi, A., Snyder, G.L., Girault, J.A., Czernik, A.J., Shao, L., Ramabhadran, R., Greengard, P., and Nairn, A.C. (2001). ARPP-16/ARPP-19: a highly conserved family of cAMP-regulated phosphoproteins. J Neurochem 77, 229-238.<br>• Dupre, A., Buffin, E., Roustan, C., Nairn, A.C., Jessus, C., and Haccard, O. (2013). The phosphorylation of ARPP19 by Greatwall renders the auto-amplification of MPF independently of PKA in Xenopus oocytes. J Cell Sci 126, 3916-3926.<br>• Fig. 8d, Fig. 9a and Supp Fig. 5a: Stable expression of Arpp19 in Xenopus oocytes during meiotic maturation.

S109-phosphorylated Arpp19:
• Dupre, A., Daldello, E.M., Nairn, A.C., Jessus, C., and Haccard, O. (2014). Phosphorylation of ARPP19 by protein kinase A prevents meiosis resumption in Xenopus oocytes. Nature Communications 5, 3318.
• Dupre, A., Haccard, O. and Jessus, C. (2017). The greatwall kinase is dominant over PKA in controlling the antagonistic function of ARPP19 in Xenopus oocytes. Cell Cycle 16, 1440-1452.
• Fig. 1a,c,e, Fig. 2b,d, Fig. 3b,c, Fig. 4a,b, Fig. 5a,b, Fig. 6d, Fig. 7a,e,g, Fig. 8a,d, Fig. 9a,c, Supp Fig. 2a,c,d, Supp Fig. 3a, Supp Fig. 4a,b and Supp Fig. 5a: Detection of Arpp19 when phosphorylated by PKA, in Xenopus oocytes, in extracts and in phosphatase and kinase assays.

S67-phosphorylated Arpp19:
• Dupre, A., Buffin, E., Roustan, C., Nairn, A.C., Jessus, C., and Haccard, O. (2013). The phosphorylation of ARPP19 by Greatwall renders the auto-amplification of MPF independently of PKA in Xenopus oocytes. J Cell Sci 126, 3916-3926.
• Dupre, A., Haccard, O. and Jessus, C. (2017). The greatwall kinase is dominant over PKA in controlling the antagonistic function of ARPP19 in Xenopus oocytes. Cell Cycle 16, 1440-1452.
• Fig 6b, Fig. 7c and Supp Fig. 3b: Analysis of Arpp19 dephosphorylation at S67 when added to prophase extracts supplemented or not with PKI, in the presence or in the absence of OA.

PP1:
• Ma, S., Vigneron, S., Robert, P., Strub, J.M., Cianferani, S., Castro, A., and Lorca, T. (2016). Greatwall dephosphorylation and inactivation upon mitotic exit is triggered by PP1. J Cell Sci 129, 1329-1339.
• Fig. 2a, Fig. 2f, Fig. 3b-c and Fig. 5a: PP1 expressed in prophase and metaphase II-arrested oocytes.

PP2A-A and PP2A-C antibodies:
• Bosch, M., Cayla, X., Van Hoof, C., Hemmings, B.A., Ozon, R., Merlevede, W., and Goris, J. (1995). The PR55 and PR65 subunits of protein phosphatase 2A from Xenopus laevis molecular cloning and developmental regulation of expression. Eur J Biochem 230, 1037-1045.
• Dupre, A., Buffin, E., Roustan, C., Nairn, A.C., Jessus, C., and Haccard, O. (2013). The phosphorylation of ARPP19 by Greatwall renders the auto-amplification of MPF independently of PKA in Xenopus oocytes. J Cell Sci 126, 3916-3926.
• Fig. 2a,f, Fig. 3b,c, Fig. 4a,b, Fig. 5a,b, Supp Fig. 2c,d and Supp Fig. 3c: PP2A-A/C expressed in prophase and metaphase II-arrested oocytes.

B55δ:
• Mochida, S., Ikeo, S., Gannon, J., and Hunt, T. (2009). Regulated activity of PP2A-B55delta is crucial for controlling entry into and exit from mitosis in Xenopus egg extracts. Embo J. 28, 2777-2785.
• Mochida, S., Maslen, S.L., Skehel, M., and Hunt, T. (2010). Greatwall Phosphorylates an Inhibitor of Protein Phosphatase 2A That Is Essential for Mitosis. Science 330, 1670-1673.
• Dupre, A., Buffin, E., Roustan, C., Nairn, A.C., Jessus, C., and Haccard, O. (2013). The phosphorylation of ARPP19 by Greatwall renders the auto-amplification of MPF independently of PKA in Xenopus oocytes. J Cell Sci 126, 3916-3926.
• Fig. 2a, Fig. 3b,c, Fig. 4a,b, Fig. 5a,b, Fig. 5a, Supp Fig. 2c,d and Supp Fig. 3c: B55δ expressed in prophase and metaphase II-arrested oocytes.

B56ε:
• Mochida, S., Ikeo, S., Gannon, J., and Hunt, T. (2009). Regulated activity of PP2A-B55delta is crucial for controlling entry into and exit from mitosis in Xenopus egg extracts. Embo J. 28, 2777-2785.
• Hino, H., Takaki, K., and Mochida, S. (2015). Inhibitor-1 and -2 of PP2A have preference between PP2A complexes. Biochem Biophys Res Commun 467, 297-302.
• Fig. 2a, Fig. 3b-c, Fig. 5a-b and Supp Fig. 3c: B56ε expressed in prophase and metaphase II-arrested oocytes.

PP4:
• Abcam Website : Goat polyclonal to PP-X (Abcam ab115741). Application: ELISA, IHC-P, WB. Reactivity: Human (predicted: Mouse, Rat, Rabbit, Cow, Xenopus laevis, Zebrafish, Orangutan, Xenopus tropicalis), Immunogen : PQETRGIPSKKP
• 100% identity of human PP4 with Xenopus PP4.
• Fig. 2a,f, Fig. 3b,c, Fig. 4a, Fig. 5a and Supp Fig. 2c: PP4 expressed in prophase and metaphase II-arrested oocytes. The antibody generates a signal at the expected molecular weight of Xenopus PP4 catalytic subunit (35.1 kDa).

PP5:
• Mochida, S., Ikeo, S., Gannon, J., and Hunt, T. (2009). Regulated activity of PP2A-B55delta is crucial for controlling entry into and exit from mitosis in Xenopus egg extracts. Embo J. 28, 2777-2785.
• Fig. 2a and 2f, Fig. 3b-c, and Fig.5a-b: PP5 expressed in prophase and metaphase II-arrested oocytes.

PKA catalytic subunit:
• Abcam Website : Anti-cAMP Protein Kinase Catalytic subunit antibody (Abcam ab26322). Rabbit polyclonal to cAMP Protein Kinase Catalytic subunit, Host species : Rabbit, Tested applications : ICC/IF, WB, IP, ICC, IHC, Species reactivity : Mouse, Rat, Sheep, Rabbit, Guinea pig, Hamster, Cow, Dog, Human, Pig, Xenopus laevis, Monkey, Immunogen : Synthetic peptide PKFKGPGDTSNFDDYEEEC conjugated to KLH, corresponding to amino acids 316-333 of Human cAMP Protein Kinase Catalytic subunit.
• Fig. 2f: PKA-C expressed in prophase-arrested oocytes.

PKI:
• Abcam Datasheet : Anti-PKI-alpha antibody (Abcam ab122816), Host species : Rabbit, Suitable for: IHC-P, ICC/IF, Reacts with: Human, Predicted to work with: Mouse, Antigen sequence: rnaihdilvs sasgnsnelalklagldinktegeedaqrs steqsgeaqg eaakses (amino acids 20-76 of Human PKI-alpha).
• Sequence alignment: 100% identity of Xenopus sequence with rat PKI
Human PKI: rnaihdilvssasgnsnelalklagldinktegeedaqrssteqsgeaqgeaakses
Rat PKI: rnaihdilvssasgnsnelalklagldinktegeddgqrssteqsgeaqgeaakses
• Fig2f: Western blot analysis of prophase extracts supplemented with PKI.

PP6:
• Bethyl website: Rabbit anti-PPP6C Antibody (Bethyl A300-844A), Affinity Purified, Reactivity : Human, Mouse, Applications : WB, IP, Host : Rabbit, Antibody Type : Polyclonal, Immunogen : between 250 and C-term, Uniprot ID O00743, 100% Sequence Identity Rat
• Sequence alignment : 100% identity of the immunogen with Xenopus PP6
Hum (Refseq : NM_001123369.1) yrcgniasim vfkdvntr epklfravpd servipprtt tpyfl
Rat (Refseq : BC078747.1) yrcgniasim vfkdvntr epklfravpd servipprtt tpyfl
Xen (Refseq : NM_001095920.1) yrcgniasim vfkdvntr epklfravpd servipprtt tpyfl
• Fig. 2a,f, Fig. 3b,c and Fig. 5a: PP6 expressed in prophase and metaphase II-arrested oocytes. The antibody produces a signal at the expected molecular weight for Xenopus PP6 catalytic subunit (34,5 kDa).

Phosphorylated MAPK:
• Cell signaling Website : reference Cell Signaling 9106, Reactivity : Human, Mouse, Rat, Hamster, Monkey, Mink, Bovine, Pig, Zebrafish, MW (kDa) : 44, Isotype : Mouse IgG1 H M R Hm Mk Mi Z B Pg,
• Daldello, E.M., Le, T., Poulhe, R., Jessus, C., Haccard, O., and Dupre, A. (2015). Fine-tuning of Cdc6 accumulation by Cdk1 and MAP kinase is essential for completion of oocyte meiotic divisions. J Cell Sci. 128, 2482-2496.
• Dupré, A., Jessus, C., Ozon, R., and Haccard, O. (2002). Mos is not required for the initiation of meiotic maturation in Xenopus oocytes. EMBO J 21, 4026-4036.
• Dupre, A., Buffin, E., Roustan, C., Nairn, A.C., Jessus, C., and Haccard, O. (2013). The phosphorylation of ARPP19 by Greatwall renders the auto-amplification of MPF independently of PKA in Xenopus oocytes. J Cell Sci 126, 3916-3926.
• Haccard, O., and Jessus, C. (2006). Redundant pathways for Cdc2 activation in Xenopus oocyte: either cyclin B or Mos synthesis. EMBO Rep 7, 321–325.
• Fig 8d and Fig. 9a: MAPK is phosphorylated at the time of Cdk1 activation in response to progesterone addition.

Total MAPK:
• Abcam Datasheet : Source : ERK 2 (Abcam SC-154) (C-14) is a rabbit affinity purified polyclonal antibody raised against a peptide mapping at the C-terminus of ERK 2 of rat origin, Application : ERK 2 (C-14) is recommended for detection of ERK 2 p42 and, to a lesser extent, ERK 1 p44 of mouse, rat, human, Xenopus laevis and zebrafish origin by Western Blotting.
• Daldello, E.M., Le, T., Poulhe, R., Jessus, C., Haccard, O., and Dupre, A. (2015). Fine-tuning of Cdc6 accumulation by Cdk1 and MAP kinase is essential for completion of oocyte meiotic divisions. J Cell Sci. 126, 3916-3926.
• Dupré, A., Jessus, C., Ozon, R., and Haccard, O. (2002). Mos is not required for the initiation of meiotic maturation in Xenopus oocytes. EMBO J 21, 4026-4036.
• Fig. 8d and Fig. 9a: The expression of MAPK is stable during meiotic maturation induced by progesterone.

Karyopherin B1:
• Datasheet: Source karyopherin B1 (SC-1863, Santa Cruz) is a goat polyclonal antibody raised againt a peptide mapping at the C-terminus of human karyopherin, also known as Importin subunit B1.
• Gaffré, M., Martoriati, A., Belhachemi, A., Chambon, JP, Houliston, E., Jessus, C., and Anthi Karaiskou (2011). A critical balance between Cyclin B synthesis and Myt1 activity controls meiosis entry in Xenopus oocytes. Development 138, 3735-3744.
• Supp Fig. 3c: The expression level of karyopherin is stable in extracts depleted or not for PP2A. The protein does not associate in a non-specific manner with the beads.

Poly-His:
• Sigma Datasheet: Anti-polyHistidine monoclonal antibody (Sigma A7058) is a mouse IgG2a isotype derived from the HIS-1 hybridoma produced by the fusion of mouse myeloma cells and splenocytes from a mouse immunized with a polyhistidine-tagged fusion protein.
• Fig. 6a: Detection of His-tagged proteins translated in oocytes after mRNA injection (PP1 and B55δ).

GST:
• Sigma Datasheet: Anti-Glutathione-S-Transferase (GST) polyclonal antibody (Sigma A-7340) is produced in rabbit using repeated injections of recombinant GST from Schistosoma japonicum expressed in E. coli as the immunogen.
• Fig. 1a,c,e, Fig. 2b,d, Fig. 3b,c, Fig. 4a,b, Fig. 5a,b, Fig. 6b,d, Fig. 7a,c,e,g, Fig. 8a,d, Fig. 9c, Supp Fig. 2a,c,d, Supp Fig. 3a,b and Supp Fig. 4a,b,d: Detection of GST-tagged Arpp19 added in oocyte extracts, or injected into oocytes, or used as a substrate in phosphatase and kinase assays.

Animals and other organisms

Policy information about studies involving animals; ARRIVE guidelines recommended for reporting animal research

Laboratory animals	No live animals were used in the study. Full-grown prophase oocytes are obtained from unprimed Xenopus laevis adult females.
Wild animals	The study does not involve wild animals.
Field-collected samples	Samples were not collected from the field.
Ethics oversight	The research proposal requires the use of Xenopus laevis (referred to as animal) and takes place at the IBPS (Institute of Biology Paris-Seine) aquatic facility in Paris, France. According to the Directive 2010/63/EU, care and maintenance of Xenopus laevis in this facility follow European legislation (EU Directive 2010/63/EU). The IBPS aquatic facility and the project received official approval from the French Ministry of Agriculture to carry out research and experiments on animals: Animal Facility agreement #A 75-05-25 and scientific project approval #APAFIS#14127-2018031614373133v2. The experiments on animals are conducted by authorised personnel only.

Note that full information on the approval of the study protocol must also be provided in the manuscript.
